# Effects of Selected Prebiotics or Synbiotics Administered in ovo on Lymphocyte Subsets in Bursa of the Fabricius, Thymus, and Spleen in Non-Immunized and Immunized Chicken Broilers

**DOI:** 10.3390/ani11020476

**Published:** 2021-02-11

**Authors:** Marianna Szczypka, Agnieszka Suszko-Pawłowska, Maciej Kuczkowski, Michał Gorczykowski, Magdalena Lis, Artur Kowalczyk, Ewa Łukaszewicz, Dominik Poradowski, Iwona Zbyryt, Marek Bednarczyk, Tadeusz Stefaniak

**Affiliations:** 1 Department of Pharmacology and Toxicology, Faculty of Veterinary Medicine, Wrocław University of Environmental and Life Sciences, Norwida 31, 50-375 Wrocław, Poland; agnieszka.suszko@upwr.edu.pl (A.S.-P.); magdalena.lis@upwr.edu.pl (M.L.); 2 Department of Epizootiology and Clinic of Bird and Exotic Animals, Faculty of Veterinary Medicine, Wrocław University of Environmental and Life Sciences, Pl. Grunwaldzki 45, 50-366 Wrocław, Poland; maciej.kuczkowski@upwr.edu.pl (M.K.); iwona.zbyryt@upwr.edu.pl (I.Z.); 3 Department of Internal Medicine and Clinic of Diseases of Horses, Dogs and Cats, Division of Parasitology, Faculty of Veterinary Medicine, Wrocław University of Environmental and Life Sciences, Norwida 31, 50-375 Wrocław, Poland; michal.gorczykowski@upwr.edu.pl; 4 Institute of Animal Breeding, Division of Poultry Breeding, Wrocław University of Environmental and Life Sciences, Chełmońskiego 38c, 51-630 Wrocław, Poland; artur.kowalczyk@upwr.edu.pl (A.K.); ewa.lukaszewicz@upwr.edu.pl (E.Ł.); 5 Department of Animal Physiology and Biostructure, Division of Animal Anatomy, Faculty of Veterinary Medicine, Wrocław University of Environmental and Life Sciences, Kożuchowska 1, 51-631 Wrocław, Poland; dominik.poradowski@upwr.edu.pl; 6 Department of Animal Biotechnology and Genetics, UTP University of Science and Technology, Mazowiecka 28, 85-084 Bydgoszcz, Poland; marbed13@op.pl; 7 Department of Immunology, Pathophysiology and Veterinary Preventive Medicine, Faculty of Veterinary Medicine, Wrocław University of Environmental and Life Sciences, Norwida 31, 50-375 Wrocław, Poland; tadeusz.stefaniak@upwr.edu.pl

**Keywords:** prebiotics, synbiotics, lymphocyte subsets, broiler

## Abstract

**Simple Summary:**

Probiotics, prebiotics, and synbiotics may be used as feed additives instead of banned antibiotic-based growth promoters. These bioactive compounds applied in ovo have beneficial effects on intestinal bifidobacteria, decrease the number of detrimental bacteria in the gut, stimulate the development of gut-associated lymphoid tissues (GALT), and modulate the development of lymphoid organs. The aim of our study was to determine whether the specific in ovo-delivered prebiotics and synbiotics affected the lymphocyte subsets of the bursa of the Fabricius, thymus, and spleen in non-immunized chicken broilers and in birds immunized with T-dependent (sheep red blood cells—SRBC) and T-independent (dextran—DEX) antigens. This study demonstrated that in ovo administration of prebiotics and synbiotics is a promising approach for enhancing chicken immune system functions. We conclude that a combination of inulin and *Lactococcus lactis* subsp. *lactis* IBB SL1 was the most effective of the tested compounds in the stimulation of the chicken immune system.

**Abstract:**

The effects of in ovo-delivered prebiotics and synbiotics on the lymphocyte subsets of the lymphoid organs in non-immunized 7-day-old broiler chickens and in non-immunized, sheep red blood cells (SRBC)-immunized, and dextran (DEX)-immunized 21- and 35-day-old birds were studied. The substances were injected on the 12th day of egg incubation: Prebiotic1 group (Pre1) with a solution of inulin, Prebiotic2 group (Pre2) with a solution of Bi^2^tos (non-digestive transgalacto-oligosaccharides), Synbiotic1 group (Syn1) with inulin and *Lactococcus lactis* subsp. *lactis* IBB SL1, and Synbiotic2 group (Syn2) with Bi^2^tos and *Lactococcus lactis* subsp. *cremoris* IBB SC1. In 7-day-old chicks, a decrease in T splenocytes was noticed in all groups. The most pronounced effect in 21- and 35-day-old birds was an increase in TCRγδ^+^ cells in Syn1 and Syn2 groups. A decrease in bursal B cells was observed in DEX-immunized Pre1 group (21-day-old birds), and in the Syn1 group in non-immunized and SRBC-immunized 35-day-old birds. An increase in double-positive lymphocytes was observed in Pre1 (35-day-old birds) and Pre2 (immunized 21-day-old birds) groups. In Pre1 and Syn1 groups (21- and 35-day-old), an increase in B splenocytes and a decrease in T splenocytes were observed. We concluded that Syn1 was the most effective in the stimulation of the chicken immune system.

## 1. Introduction

Nowadays, in the absence of antibiotic-based growth promoters used in poultry farming, there is a continuing need to search for alternatives. Novel feed additives should positively affect the host and improve its intestinal health and general performance by promoting probiotic and prebiotic effects. Probiotics are live microorganisms beneficial to the host, prebiotics are non-digestible substances that selectively stimulate growth and/or the metabolic activity of beneficial intestinal microflora, while synbiotics are defined as synergistic combinations of prebiotics with probiotic bacteria [1,2].

Both commensal intestinal bacteria and probiotic bacteria have various health benefits. Some of them prevent the growth of pathogenic bacteria by producing bacteriocins, competing with pathogens for nutrients and binding sites on mucosal surfaces, and by stimulating the immune system [2,3]. A drop in the population of pathogenic bacteria was observed after the introduction of probiotic bacteria as dietary supplementation in poultry [1,4]. Beneficial gut microbiota also play an important role in the development of the immune system by interacting with the gut-associated lymphoid tissues (GALT) [5,6].

Prebiotics and synbiotics used as feed additives in broiler chickens favour the growth of beneficial intestinal bacteria and inhibit growth of pathogenic microorganisms [1,7,8,9]. Dietary supplementation with prebiotics modulates the immune response in broiler chickens by positively affecting their commensal intestinal bacteria as microflora growth promoters [9,10]. Some studies showed that dietary supplementation with prebiotics or synbiotics may have a beneficial effect on the body weight, daily weight gain, feed conversion rate, and carcass yield percentage of broiler chickens [1,11]. However, other reports indicated no visible effects of this dietary supplementation on broiler performance [7,12,13,14]. These contradictory results may be due to different factors, such as food intake, management, experimental conditions, or unfavourable interactions of prebiotics or synbiotics with various fodder additions or drugs [7,14,15].

Positive effects of prebiotics on intestinal microbiota were observed not only after dietary supplementation, but also when these bioactive substances were administered in ovo on the 12th day of incubation [16]. In ovo technology not only eliminates the impact of the feed intake rate, but it also provides an opportunity for the earliest possible application of bioactive substances. Thus, dietary administration of prebiotics and synbiotics may be replaced by their in ovo inoculation [17,18]. An innovative method of automatic in ovo injection facilitates the use of this approach under field conditions [15]. Previous studies showed that prebiotics or synbiotics applied in ovo on the 12th day of incubation may affect the development of the central and peripheral lymphoid organs in broiler chickens and may stimulate the secondary immune response, as well as modulate leukocyte maturation and reactivity [19,20,21,22]. Chickens treated in ovo with inulin or Bi^2^tos and then immunized with sheep red blood cells (SRBC) had significantly higher serum IgG levels than the control group [22].

Our study was a part of a larger project concerning the effects of prebiotics and synbiotics on the chicken immune system after in ovo administration. The available literature contains limited information on the effects of in ovo-delivered prebiotics or synbiotics on lymphocyte subsets in chickens. The purpose of this study was to determine the effects of specific prebiotics and synbiotics on the lymphocyte subsets of the bursa of the Fabricius, thymus, and spleen in non-immunized chicken broilers and in birds immunized with T-dependent (SRBC) and T-independent (dextran—DEX) experimental antigens.

## 2. Materials and Methods

### 2.1. In Ovo Treatment

This experiment was carried out at the experimental farm of Wrocław University of Environmental and Life Sciences (Wrocław, Poland). Hatching eggs were obtained from a 32-week-old breeder flock (Ross 308). Each of them weighed approximately 60 g. The eggs were incubated in a Petersime incubator (Petersime, Zulte, Belgium), in a commercial hatchery (Drobex, Solec Kujawski, Poland). On the 12th day of the incubation, the eggs were candled, and the infertile eggs and those containing dead embryos were removed. The eggs with live embryos were randomly allotted to five experimental groups (160 eggs per group). The eggs in each group received an equal volume (200 μL) of a bioactive compound, injected in ovo into the air cell. The control group (C) was injected with physiological saline. Prebiotic 1 group (Pre1) was injected with a solution containing 1.76 mg of inulin (Sigma–Aldrich, St. Louis, MO, USA). Prebiotic 2 group (Pre2) was injected with a solution containing 0.528 mg of Bi^2^tos (Clasado Ltd., Milton Keynes, UK), i.e., commercially developed non-digestive transgalacto-oligosaccharides. Synbiotic 1 group (Syn1) received 1.76 mg of inulin and 1000 colony-forming units (CFU) of *Lactococcus lactis* subsp. *lactis* IBB SL1. Synbiotic 2 group (Syn2) received 0.528 mg of Bi^2^tos and 1000 CFU of *Lactococcus lactis* subsp. *cremoris* IBB SC1.

The bacterial cultures were prepared as follows: the number of bacteria in a fresh over-night culture of IBB SL1 and IBB SC1 strains was estimated at 3 × 10^8^ of living cells/mL in GM17 liquid medium. The final bacterial suspension of 1000 CFU in 20 μL was obtained by diluting the bacterial cultures with a prebiotic solution immediately prior to the injection. The synbiotics injected in the Syn1 and Syn2 groups consisted of 180 μL of the prebiotic solution and 20 μL of the bacterial suspension. The punctures in the egg shell were sealed using a special automatic system [15], and the incubation was continued until hatching.

### 2.2. Post Hatch Treatment of Animals

The experiments were carried out using male broiler chickens hatched from the injected eggs. The rearing conditions were described by Stefaniak et al. [21]. The feed was free from antibiotics, probiotics, and prebiotics.

The study involved 7-, 21,- and 35-day-old male broiler chickens. The chickens from the experimental groups of 7-day-old birds were not immunized. There were three subgroups within 21 and 35-day-old birds: non-immunized, SRBC-immunized, and DEX-immunized. The study protocol was approved by the Local Ethics Committee in Bydgoszcz (No. 24/2011). The total count of birds used in this experiment was 245. The schedule of the experiment is presented in Table 1.

### 2.3. Immunization

On day 7, chickens from each of the experimental groups (C, Pre1, Pre2, Syn1, and Syn2) were randomly allocated into three subgroups: (1) non-immunized birds, (2) SRBC-immunized birds (SRBC) that were treated intramuscularly with 200 μL of 5% suspension of SRBC (Pro Animali, Wrocław, Poland), (3) dextran-immunized birds (DEX) that were immunized subcutaneously with 1 mg of dextran (molecular weight 5–40 MDa; Koch Light Laboratories, Ltd., Haverhill, UK) dissolved in 200 μL of PBS. These immunization procedures were repeated in some groups on day 21. Thus, the chickens euthanized on the 21st day were immunized once on the 7th day and the birds euthanized on the 35th day were immunized twice, on the 7th and 21st days. The immunization scheme and the count of immunized chickens were described by Stefaniak et al. [22].

### 2.4. Measurements

The chickens were slaughtered by cervical dislocation. In 7-day-old chicks, the bursa of Fabricius and spleen were immediately pulled out. In 21- and 35-day-old birds the bursa of the Fabricius, thymus, and spleen were removed. These organs were placed in Petri dishes containing sterile, ice-cold phosphate-buffered saline solution (PBS) (Institute of Immunology and Experimental Therapy, Polish Academy of Sciences, Wrocław, Poland). Cells were released from the lymphoid organs by a passage through a nylon mesh. Then, the cell suspensions were layered 1:1 on the isolation mixture Histopaque (Sigma–Aldrich, St. Louis, MO, USA). After centrifugation (3000× *g*, 15 min, 4 °C), the cells were collected from the interphase, washed, and centrifuged (380× *g*, 8 min, 4 °C) twice with 4 °C PBS supplemented with 1% bovine serum albumin (BSA) (Sigma–Aldrich).

The cells in the suspension were stained with monoclonal antibodies at the concentrations recommended by the manufacturer. The following monoclonal antibodies (SouthernBiotech, Birmingham, AL, USA) were used in the experiment: Mouse Anti-Chicken Bu-1-FITC (Clone AV20), Mouse Anti-Chicken CD3-R-PE (Clone CT-3), Mouse Anti-Chicken TCRγδ-FITC (Clone TCR-1), Mouse Anti-Chicken CD4-FITC (Clone CT-4), and Mouse Anti-Chicken CD8α-CY-5 (Clone CT-8).

The lymphocytes were incubated with the following antibodies: the bursal lymphocytes with anti-Bu-1 and anti-CD3 antibodies, the thymocytes with anti-CD3, anti-TCRγδ, anti-CD4, and anti-CD8 antibodies, and the splenocytes with anti-Bu-1, anti-CD3, anti-TCRγδ, anti-CD4, and anti-CD8 antibodies. The determined subsets of CD3^+^ splenocytes included TCRγδ^+^, CD4^+^, and CD8^+^ cells. CD subsets and the CD4^+^/CD8^+^ ratio were determined. The experiments also included staining with appropriate isotype controls.

After incubation at 4 °C for 30 min, the cells were washed and centrifuged (380× *g*, 8 min, 4 °C) twice with ice-cold PBS. Fluorescence was analyzed using a flow cytometer (BD FACSCalibur, BD Biosciences, San Jose, CA, USA). Lymphocyte marker distribution was analyzed using CellQuest 3.1f. Pro software (Becton Dickinson, San Jose, CA, USA). The percentage of lymphocyte subsets as well as the total lymphocyte count of each subset was determined.

### 2.5. Statistical Analysis

The data obtained in this study were analysed statistically using one-way analysis of variance (ANOVA), followed by Tukey’s post hoc test for multiple comparisons. The results were classified as statistically significant at *p* < 0.05.

## 3. Results

All data with significant differences between experimental groups are presented in the tables (as Supplementary Materials). The figures present only selected parameters and significant differences as compared with the control group.

### 3.1. The Effects of Prebiotics and Synbiotics on the Subsets of Bursal Lymphocytes 

**Figure 1:**
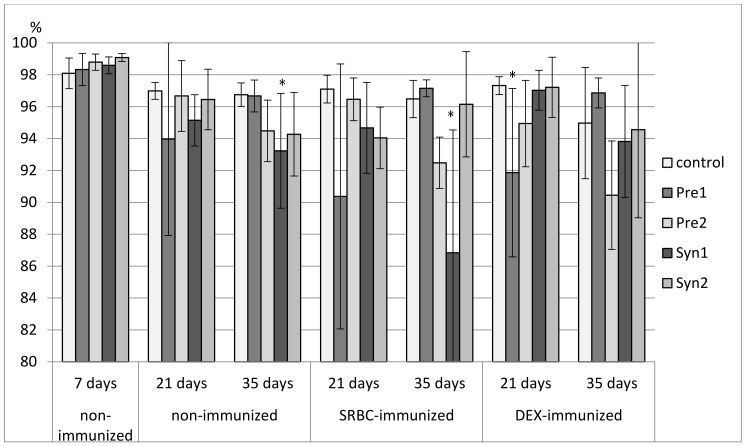
The percentage of Bu-1^+^ cells in the bursa of the Fabricius of chicken broilers after in ovo injection of the investigated prebiotics or synbiotics. The data are expressed as the mean ± standard deviation (SD). Data with a symbol (*) denote a significant difference with respect to the control group (*p* < 0.05).

In 7-day-old chicks, the percentage of bursal Bu-1^+^ lymphocytes was not affected by the in ovo treatment with investigated prebiotics and synbiotics. An increase in the percentage of CD3^+^ lymphocytes was noticed in the Syn2 group as compared with the control and Syn1 groups (Figure 1, Table S1).

Neither prebiotics nor synbiotics changed the percentage of B and T lymphocytes in 21-day-old non-immunized and SRBC-immunized chickens. In DEX-immunized 21-day-old chickens, a decrease in the percentage of Bu-1^+^ cells was observed in the Pre1 group as compared with the C and Syn1 group with an unchanged percentage of CD3^+^ cells. The tested prebiotics and synbiotics did not affect the total count of bursal lymphocyte subsets in 21-day-old birds (Figure 1, Tables S2 and S3).

In non-immunized 35-day-old chickens, Syn1 decreased the percentage of B lymphocytes as compared with the C group, but it did not influence CD3^+^ bursal lymphocytes. A similar effect was observed in SRBC-immunized 35-day-old birds, where a decrease in the percentage of Bu-1^+^ cells was noticed in the Syn1 group as compared with the C, Pre1, and Syn2 groups. This effect was accompanied by an increase in the percentage of CD3^+^ cells as compared with the Pre1 and Syn2 groups. Syn1 also boosted the total count of CD3^+^ cells as compared with the C, Pre1, and Syn2 groups. There were no changes in the percentage and total count of bursal lymphocytes in DEX-immunized 35-day-old birds (Figure 1, Tables S4 and S5).

### 3.2. The Effects of Prebiotics and Synbiotics on the Subsets of Thymocytes 

**Figure 2:**
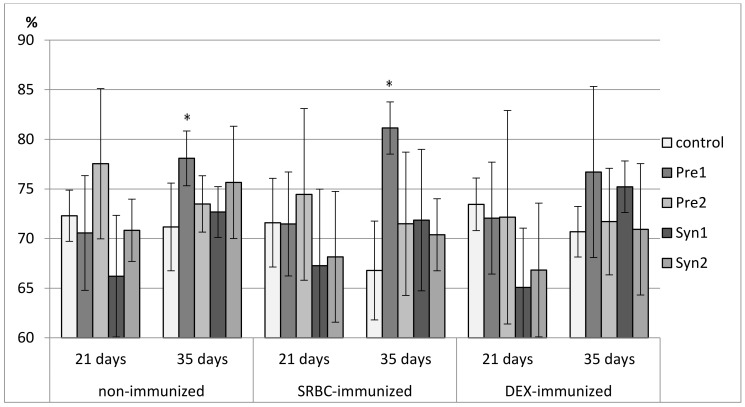
The percentage of CD4^+^CD8^+^ cells in the thymus of chicken broilers after in ovo injection of the investigated prebiotics or synbiotics. The data are expressed as the mean ± SD. Data with a symbol (*) denote a significant difference with respect to the control group (*p* < 0.05).

**Figure 3:**
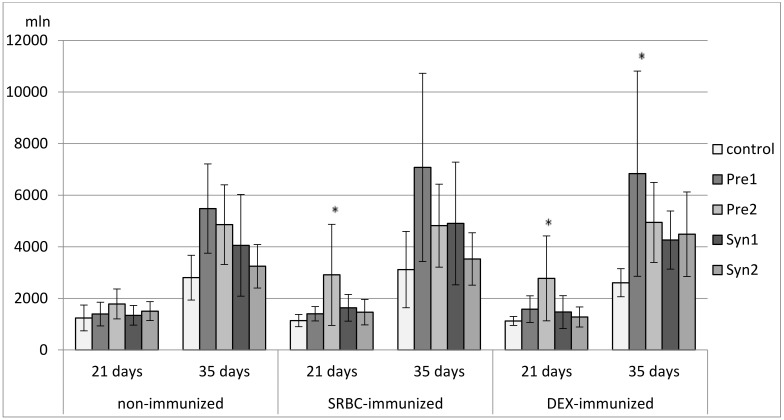
The total count of CD4^+^CD8^+^ cells in the thymus of chicken broilers after in ovo injection of the investigated prebiotics or synbiotics. The data are expressed as the mean ± SD. Data with a symbol (*) denote a significant difference with respect to the control group (*p* < 0.05).

**Figure 4:**
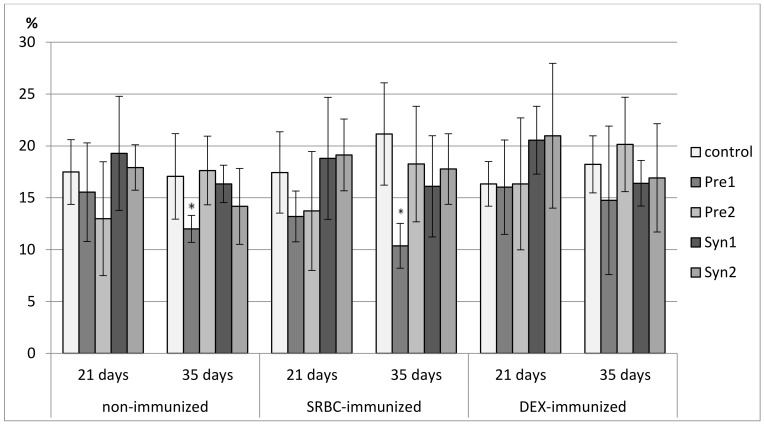
The percentage of CD4^−^CD8^−^ cells in the thymus of chicken broilers after in ovo injection of the investigated prebiotics or synbiotics. The data are expressed as the mean ± SD. Data with a symbol (*) denote a significant difference with respect to the control group (*p* < 0.05).

**Figure 5:**
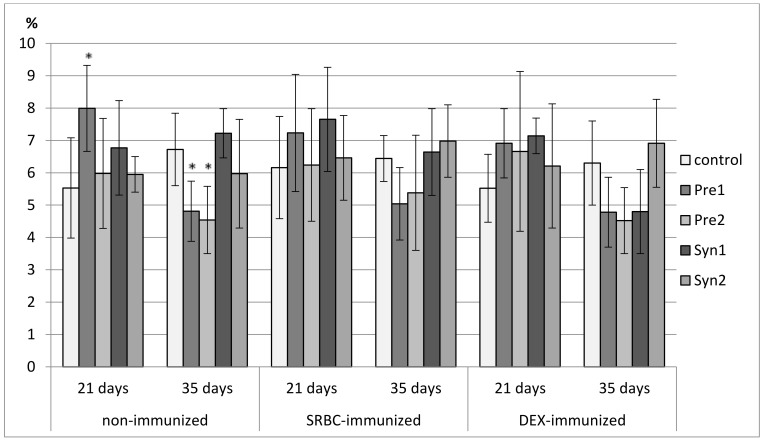
The percentage of CD8^+^ cells in the thymus of chicken broilers after in ovo injection of the investigated prebiotics or synbiotics. The data are expressed as the mean ± SD. Data with a symbol (*) denote a significant difference with respect to the control group (*p* < 0.05).

**Figure 6:**
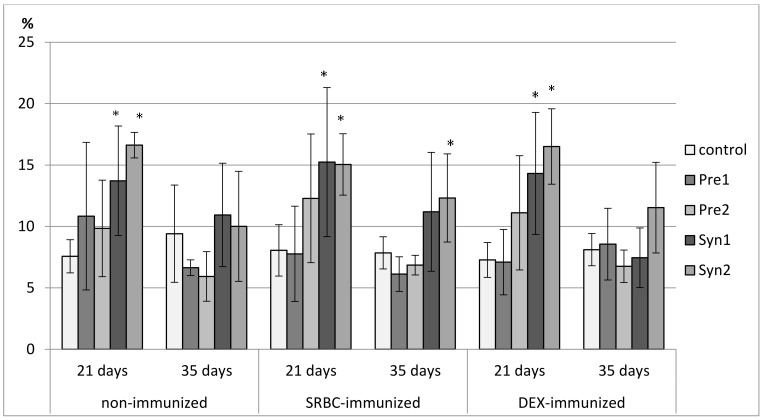
The percentage of CD3^+^TCRγδ^+^ cells in the thymus of chicken broilers after in ovo injection of the investigated prebiotics or synbiotics. The data are expressed as the mean ± SD. Data with a symbol (*) denote a significant difference with respect to the control group (*p* < 0.05).

Both in non-immunized and immunized 21-day-old chickens, Syn1 and Syn2 enhanced the percentage of CD3^+^TCRγδ^+^ lymphocytes as compared with the C group. In the immunized birds this effect was statistically significant also with the Pre1 group. In the non-immunized birds, an increase in the percentage of CD8^+^ cells was observed in the Pre1 group as compared with the C group. The percentage of double-positive thymocytes dropped in the Syn1 group in comparison with the Pre2 group. Both in SRBC- and DEX-immunized 21-day-old chickens, Pre2 increased the total count of CD4^+^CD8^+^ cells as compared with the C group. In DEX-immunized birds, an increase in the total count of CD8^+^ thymocytes was also observed (statistically significant vs. C group) (Figure 2, Figure 3, Figure 4, Figure 5 and Figure 6, Tables S6 and S7).

In non-immunized and SRBC-immunized 35-day-old chickens, Pre1 reduced the percentage of double-negative thymocytes and increased the percentage of double-positive cells. Both Pre1 and Pre2 caused a drop in the percentage of single-positive CD8^+^ cells in non-immunized 35-day-old birds. In the same birds, Syn1 decreased CD4^+^/CD8^+^ ratio as compared with the Pre1 and Pre2 groups. In SRBC-immunized birds, the percentage of CD3^+^TCRγδ^+^ was statistically higher in Syn1 and Syn2 groups than in the Pre1 group. In Syn2 group, this increase was also statistically significant as compared with the C and Pre2 groups. In non-immunized and SRBC-immunized 35-day-old birds, prebiotics and synbiotics did not affect the total cell count of lymphocyte subsets (Figure 2, Figure 3, Figure 4, Figure 5 and Figure 6, Tables S8 and S9).

In DEX-immunized 35-day-old chickens Syn2 increased the percentage of CD3^+^TCRγδ^+^ and CD4^+^ thymocytes as compared with the Pre2 and Syn1 groups as well as the percentage of CD8^+^ cells as compared with the Pre2 group. Pre1 increased the total count of CD3^+^TCRγδ^+^ and CD4^+^CD8^+^ cells as compared with the C group (Figure 2, Figure 3, Figure 4, Figure 5 and Figure 6, Tables S8 and S9).

### 3.3. The Effects of Prebiotics and Synbiotics on the Subsets of Splenocytes 

**Figure 7:**
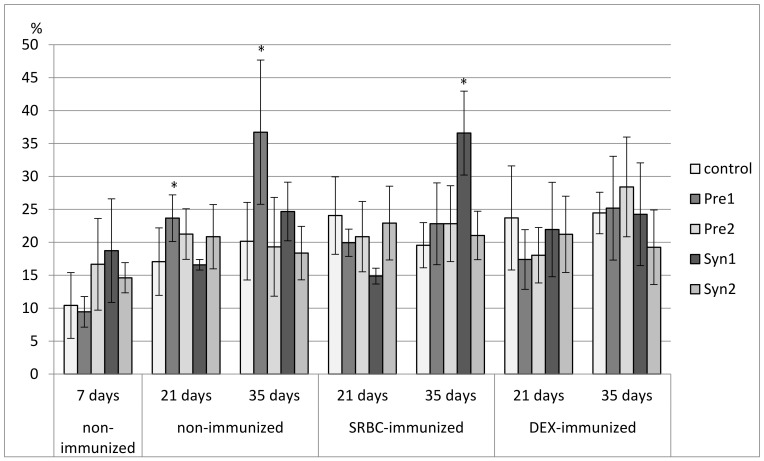
The percentage of Bu-1^+^ cells in the spleen of chicken broilers after in ovo injection of the investigated prebiotics or synbiotics. The data are expressed as the mean ± SD. Data with a symbol (*) denote a significant difference with respect to the control group (*p* < 0.05).

**Figure 8:**
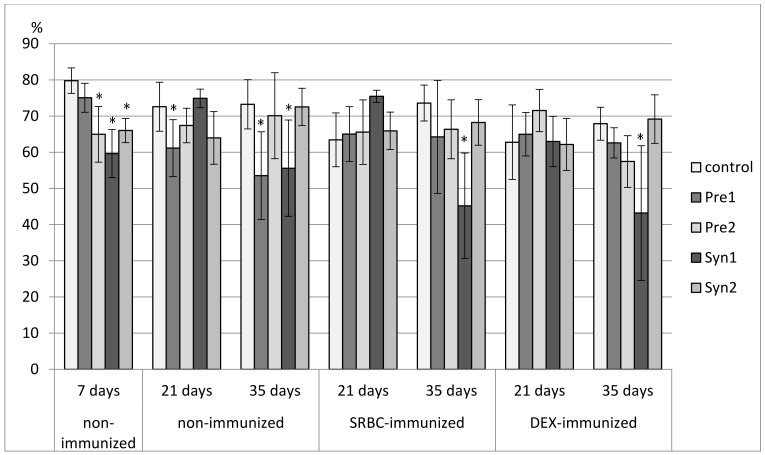
The percentage of CD3^+^ cells in the spleen of chicken broilers after in ovo injection of the investigated prebiotics or synbiotics. The data are expressed as the mean ± SD. Data with a symbol (*) denote a significant difference with respect to the control group (*p* < 0.05).

**Figure 9:**
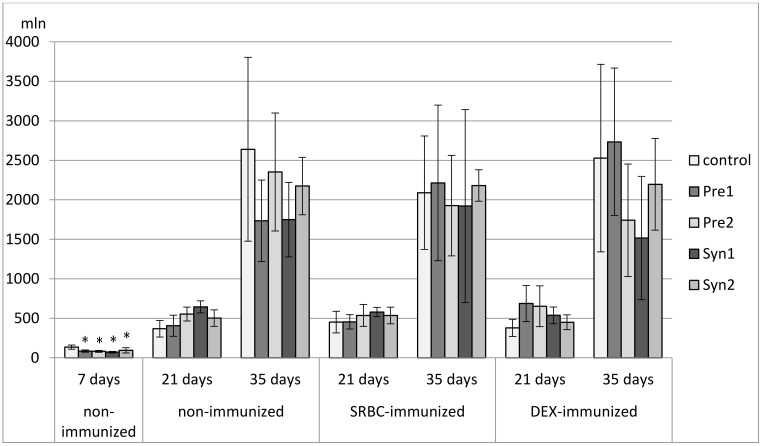
The total count of CD3^+^ cells in the spleen of chicken broilers after in ovo injection of the investigated prebiotics or synbiotics. The data are expressed as the mean ± SD. Data with a symbol (*) denote a significant difference with respect to the control group (*p* < 0.05).

**Figure 10:**
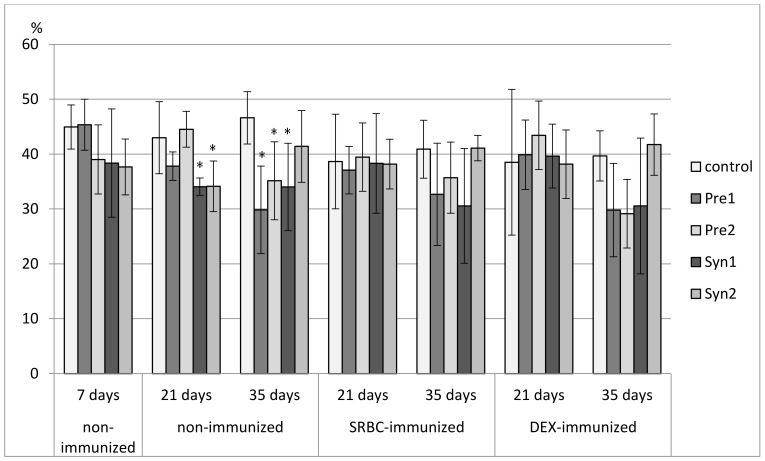
The percentage of CD8^+^ cells in the spleen of chicken broilers after in ovo injection of the investigated prebiotics or synbiotics. The data are expressed as the mean ± SD. Data with a symbol (*) denote a significant difference with respect to the control group (*p* < 0.05).

**Figure 11:**
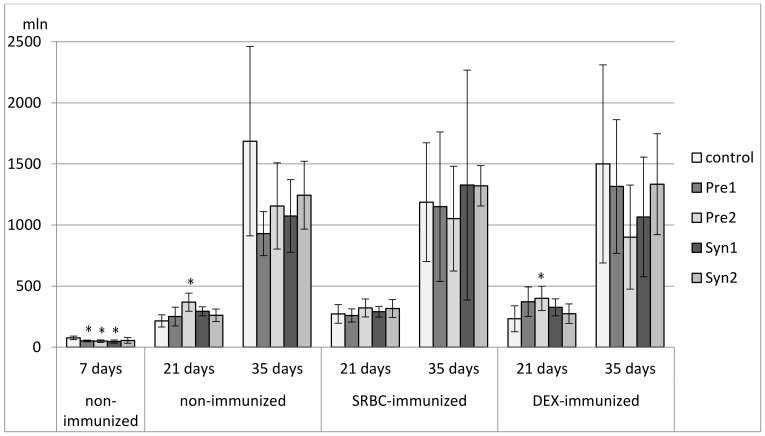
The total count of CD8^+^ cells in the spleen of chicken broilers after in ovo injection of the investigated prebiotics or synbiotics. The data are expressed as the mean ± SD. Data with a symbol (*) denote a significant difference with respect to the control group (*p* < 0.05).

**Figure 12:**
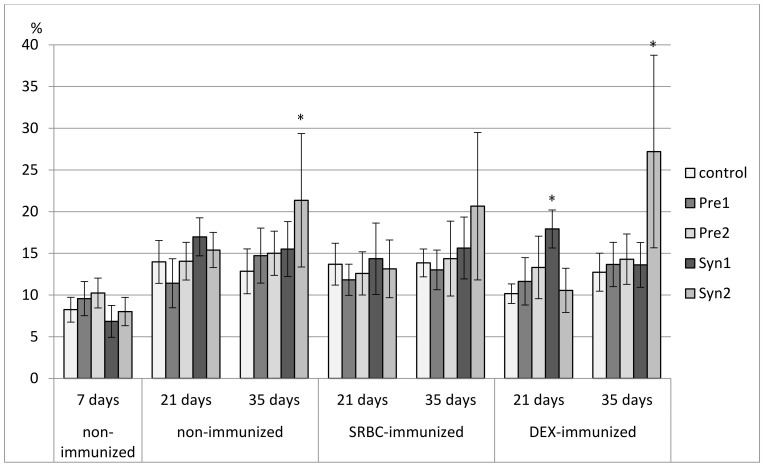
The percentage of CD3^+^TCRγδ^+^ cells in the spleen of chicken broilers after in ovo injection of the investigated prebiotics or synbiotics. The data are expressed as the mean ± SD. Data with a symbol (*) denote a significant difference with respect to the control group (*p* < 0.05).

**Figure 13:**
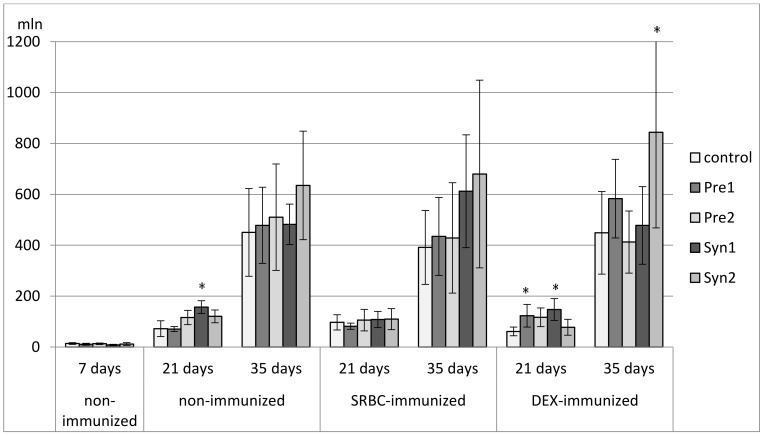
The total count of CD3^+^TCRγδ^+^ cells in the spleen of chicken broilers after in ovo injection of the investigated prebiotics or synbiotics. The data are expressed as the mean ± SD. Data with a symbol (*) denote a significant difference with respect to the control group (*p* < 0.05).

In 7-day-old chicks, a decrease in the percentage of CD3^+^ splenocytes was found in the Pre2, Syn1, and Syn2 groups as compared with the C and Pre1 groups. A decrease in total count of CD3^+^ splenocytes was observed in all tested groups. The percentage of TCRγδ^+^ cells was reduced in the Syn1 group as compared with the Pre2 group. The percentage of CD4^+^ cells was lower in the Syn1 group than in the control. None of the investigated prebiotics and synbiotics changed the percentage of CD8^+^ splenocytes or the CD4^+^/CD8^+^ ratio. A drop in the total count of CD4^+^ and CD8^+^ splenocytes was observed in the Pre1, Pre2, and Syn1 groups as compared with the control group. As for Bu-1^+^ splenocytes, an increase in their percentage was seen in the Syn1 group vs. Pre1 group (Figure 7, Figure 8, Figure 9, Figure 10, Figure 11, Figure 12 and Figure 13, Tables S10 and S11). 

In non-immunized 21-day-old chickens, Pre1 increased the percentage of B lymphocytes (Bu-1^+^ cells) with an accompanying drop in the percentage of T lymphocytes (CD3^+^ cells) as compared with the C group. The percentage of CD3^+^TCRγδ^+^ splenocytes rose in the Syn1 group as compared with the Pre1 group. Syn1 also augmented the total count of CD3^+^TCRγδ^+^ cells as compared with the C and Pre1 groups. Syn1 and Syn2 reduced the percentage of CD3^+^CD8^+^ splenocytes as compared with the C and Pre2 groups. An increase in the total count of CD3^+^CD8^+^ cells was observed in the Pre2 group as compared with the C group (Figure 7, Figure 8, Figure 9, Figure 10, Figure 11, Figure 12 and Figure 13, Tables S12 and S13).

The tested compounds did not affect the percentage and total count of splenocyte subsets in SRBC-immunized 21-day-old birds (Figure 7, Figure 8, Figure 9, Figure 10, Figure 11, Figure 12 and Figure 13, Tables S12 and S13).

In DEX-immunized 21-day-old chickens, Syn1 augmented the percentage of CD3^+^TCRγδ^+^ splenocytes as compared with the C, Pre1, and Syn2 groups. Pre1 and Syn1 increased the total count of CD3^+^TCRγδ^+^ cells as compared with the C group. Pre2 boosted the total count of CD3^+^CD8^+^ splenocytes as compared with the C group (Figure 7, Figure 8, Figure 9, Figure 10, Figure 11, Figure 12 and Figure 13, Tables S12 and S13).

In 35-day-old non-immunized chickens, an increase in the percentage of Bu-1^+^ cells was observed in the Pre1 group as compared with all other groups. A rise in the total count of Bu-1^+^ cells was noticed in the Pre1 group as compared with the Syn2 group. As for T lymphocytes (CD3^+^ cells), a decrease in their percentage was found in the Pre1 group as compared with the C, Pre2 and Syn2 groups. Syn1 also curbed the percentage of CD3^+^ cells as compared with the C and Syn2 groups. The percentage of CD3^+^CD8^+^ cells was lower in the Pre1, Pre2, and Syn1 groups than in the C group. Syn2 increased the percentage CD3^+^TCRγδ^+^, and Pre2 increased the CD4^+^/CD8^+^ ratio as compared with the C group (Figure 7, Figure 8, Figure 9, Figure 10, Figure 11, Figure 12 and Figure 13, Tables S14 and S15).

In 35-day-old SRBC-immunized chickens, Syn1 increased the percentage of Bu-1^+^ cells with an accompanying decrease in the percentage of T lymphocytes (CD3^+^ cells and CD3^+^CD4^+^ cells). These changes were statistically significant as compared with all other groups. Syn1 also enhanced the total count of B splenocytes as compared with the C, Pre2, and Syn2 groups. The ratio of CD4^+^/CD8^+^ was statistically lower in Syn1 than in the Pre1 group (Figure 7, Figure 8, Figure 9, Figure 10, Figure 11, Figure 12 and Figure 13, Tables S14 and S15).

In 35-day-old DEX-immunized chickens, a reduction in the percentage of CD3^+^ cells was observed in the Syn1 group as compared with the C, Pre1, and Syn2 groups. Syn2 increased the percentage (as compared with all other groups) and total count of CD3^+^TCRγδ^+^ cells (as compared with the C and Pre2 groups). Syn2 also augmented the percentage of CD3^+^CD8^+^ cells as compared with the Pre2 group (Figure 7, Figure 8, Figure 9, Figure 10, Figure 11, Figure 12 and Figure 13, Tables S14 and S15).

## 4. Discussion

In our study, prebiotics and synbiotics were administered in ovo on the 12th day of egg incubation We chose that day as the optimal time for this type of injection according to published studies and the immunophysiology of the chicken gut. Villaluenga et al. [18] conducted a study in which prebiotics were injected on the 1st, 8th, 12th, and 17th day of incubation. Then, the number of bifidobacteria in the faeces of 2-day-old chicks was determined. It was found that all investigated oligosaccharides increased the number of bifidobacteria, but the greatest increase was observed when the prebiotics were administered on the 12th day of embryogenesis [18]. Likewise, Pilarski et al. [17] reported that enhanced concentration of intestinal bifidobacteria in the faeces after a single in ovo injection of prebiotics on the 12th day of incubation was maintained for six weeks. Furthermore, Sławińska et al. [23] administered in ovo two *Lactococcus lactis* strains with raffinose family oligosaccharides (RFOs) prebiotics (as synbiotics) on the 12th day of embryogenesis and confirmed survivability of both bacterial strains in the chicken guts for 42 days. Moreover, the 12th day of egg incubation is one of the important times in the development of the bird immune system [24]. For example, at this time, there is a second wave of colonization of the developing thymus epithelium by T cell progenitors from para-aortic foci. Also, on the 12th day of incubation, in the spleen, basal expression levels of chicken cytokines (IFN-γ, IL-4, IL-10, and IL-18) have been identified [24]. Additionally, after this term, the allantochorion is completely developed and highly vascularized and serves as an efficient transport route from egg air cells to the amniotic fluid and the blood [18]. Taking into consideration both the development of the immune system and the bacterial colonization of the chicken guts, the 12th day of egg incubation seems to be the optimal time for in ovo injection of prebiotics and synbiotics.

In the present study, the effects of in ovo-delivered prebiotics and synbiotics on lymphocyte subsets were determined in non-immunized and immunized chicken broilers. The most pronounced change was an increase in the subsets of CD3^+^TCRγδ^+^ cells evoked by inulin and *Lactococcus lactis* subsp. *lactis* (Syn1) and Bi^2^tos and *Lactococcus lactis* subsp. *cremoris* (Syn2). This effect was observed in the thymus in non-immunized and immunized 21-day-old chickens. On day 35, this effect was maintained only in the SRBC-immunized Syn2 group. This rise was also noticed in the spleen in the non-immunized and DEX-immunized 21-day-old Syn1 group, the DEX-immunized 21-day-old Pre1 group, and in the non-immunized and DEX-immunized 35-day-old Syn2 group. TCRγδ^+^ T cells played a role in the immune response to pathogens or immunization that was demonstrated in the study using non-attenuated and live-attenuated *Salmonella enterica* strains [25] or commercial killed *Salmonella* Enteritidis vaccine [26], as well as in chickens with infectious bronchitis virus (IBV) infection [27]. Therefore, these stimulatory effects of the tested synbiotics on CD3^+^TCRγδ^+^ T cell subsets may be highly valuable, considering the immune response to pathogens or vaccinations.

The bursa of Fabricius is the primary lymphatic organ in birds, responsible for the development of B lymphocytes. In birds, B lymphocytes can be distinguished by the expression of the Bu-1 antigen (also referred to as the chB6 antigen) [28]. In our study, a decrease in Bu-1^+^ bursal lymphocytes was observed in the DEX-immunized 21-day-old Pre1 group. The same changes were noticed in the Syn1 group in non-immunized and SRBC-immunized 35-day-old birds. However, in 7-day-old birds, the bursal Bu-1^+^ lymphocytes remained unaffected by the investigated prebiotics and synbiotics. This is in accordance with the study of Sato et al. [29], who reported that Bu-1 mRNA expression in the bursa of Fabricius in 1, 3, and 7-day-old chicks fed immunobiotic lactic acid bacteria-supplemented diets was unchanged.

In our study, in the spleen, a peripheral lymphoid organ, an increase in B lymphocytes was observed in non-immunized 21- and 35-day-old Pre1 groups and in the SRBC-immunized 35-day-old Syn1 group. These effects demonstrated that inulin, both as a prebiotic (Pre1) and as a synbiotic with *Lactococcus lactis* subsp. *Lactis* (Syn1), exerted more intense effects on B cells than Bi^2^tos (non-digestive transgalacto-oligosaccharides). B cells, by secreting antibodies, are the component of humoral immunity. The effects of the same prebiotics and synbiotics, with the same experimental schedule and application as in the present study, on the humoral immune response were studied by Stefaniak et al. [22]. They found significantly higher serum IgG levels in SRBC-immunized 35-day-old Pre1- and Pre2 chickens than in the control group. However, there were no significant differences regarding the level of the anti-dextran IgM and IgG antibodies between the tested groups [22]. Thus, the results obtained in our study were only partially similar (regarding Pre1 group) to the findings of Stefaniak et al. [22]. Our observations concerning the stimulatory effects of inulin on B cells are in accordance with the study of Nabizadeh [30], who showed that inulin supplementation at different doses significantly increased total IgG in treated broiler chickens. Our results also confirmed the effects of dietary inulin and *Lactobacillus* (BCRC 16092) on broilers described by Wu et al. [31]. Apart from beneficial effects on growth performance and intestinal microbiota, inulin and *Lactobacillus* supplementation increased the serum concentration of IgG and IgA [31]. Similarly, a rise in plasma titres of IgM and IgG was observed by Janardhana et al. [10] in fructo-oligosaccharide (FOS)-treated birds. These authors examined the impact of two prebiotics, mannan-oligosaccharide (MOS) and FOS, used for 25 days as dietary supplements, on the immune cells in cecal tonsils in chickens. However, contrary to our study, they found that the prebiotics reduced the percentage of B lymphocytes (without changes in the percentage of T cells) [10]. On the other hand, Kim et al. [9] reported that plasma IgA and IgG concentrations were not affected by dietary supplementation with FOS and MOS prebiotics.

The present study showed that the most pronounced effect on thymocyte maturation was observed in the Pre1 and Pre2 groups. An increase in double-positive subsets was noticed in SRBC- and DEX-immunized 21-day-old birds treated in ovo with Pre2. The same effects were observed in the Pre1 group in non-immunized and immunized 35-day-old birds with an accompanying decrease in double-negative cells. These effects may indicate acceleration of thymocyte maturation by the tested prebiotics. There is no available information concerning thymocyte subsets after prebiotic or synbiotic administration in chickens. However, there are studies on the impact of prebiotics and synbiotics on thymus morphology. Sławińska et al. [23] demonstrated that in ovo-delivered synbiotics containing raffinose family oligosaccharides (RFO) increased the density of thymocytes in the thymus cortex. Madej et al. [20], in a study with the same compounds we used in our research, demonstrated that between day 7 and 21, the cortex/medulla diameter ratio in the thymus decreased in the prebiotic and synbiotic groups as compared with the control group, and this effect may indicate an increase in thymocyte maturation.

In our study, a decrease in T splenocytes was observed in 7-day-old birds in all tested groups. A drop in CD3^+^ splenocytes was also noticed in non-immunized Pre1 groups (21- and 35-day-old) and 35-day-old Syn1 groups, both non-immunized and immunized. Contrary to our findings, Sato et al. [29] observed no changes in the level of CD3 mRNA expression in the foregut after dietary probiotic supplementation in 1- and 7-day-old chicks. An increase in CD3 mRNA expression was found only in one experimental group of 3-day-old birds [29].

In our research, comparing CD4^+^ and CD8^+^ subsets, CD8^+^ cells were more sensitive to the prebiotics and synbiotics in 21- and 35-day-old birds. However, these effects were different (an increase or a decrease), depending on the type and number of immunizations, lymhphoid organ, and type of the tested compound. In 7-day-old chicks, a decrease both in CD8^+^ and CD4^+^ splenocytes was found. Stefaniak et al. [22] studied the effects of the tested prebiotics and synbiotics on delayed-type hypersensitivity (DTH) skin reaction in broiler chickens, which is mediated by CD8^+^ cytotoxic T cells. They demonstrated that inulin (Pre1) in 7-day-old chicks inhibited DTH skin reaction. On the other hand, in the same study, Bi^2^tos (Pre2) transiently stimulated the DTH reaction in 21-day-old birds. In accordance with this effect, our study demonstrated Pre2 induced an increase in the total count of CD8^+^ splenocytes in 21-day-old birds. Considering these results, we may conclude that Bi^2^tos may stimulate cellular immune responses.

The available literature contains limited information on the effects of probiotics, prebiotics or synbiotics on the lymphocyte subsets in chickens. Madej et al. [32] reported the effects of a prebiotic (galacto-oligosaccharides), probiotic (*Lactococcus lactis* subsp. *cremoris* IBB477), and synbiotic (combination of galactooligosaccharides and *L. lactis*) delivered in ovo on day 12 of egg incubation on the number of B and T cells in the spleen and cecal tonsils (using immunohistochemistry). In broilers, on day 21, galacto-oligosaccharides increased the number of CD8^+^ splenocytes. On day 42, the synbiotic stimulated the colonization of the spleen both by B cells (Bu-1^+^) and T cells (CD4^+^ and CD8^+^ cells). Alizadeh et al. [33] investigated the effects of various in ovo-delivered doses of a multi-strain lactobacilli mixture on the innate and adaptive immune responses in broiler chickens. They demonstrated that lactobacilli administration increased the splenic expression of cytokines (IFN-α, IFN-β, IFN-γ, IL-8, and IL-12). However, the performed treatment did not affect the percentage of CD4^+^ and CD8^+^ splenocytes. There are more studies on the effects of pro-, pre- or synbiotics on the lymphocyte activity. Flaujac Lafontaine et al. [34] reported the effects of prebiotic galacto-oligosaccharide (GOS) on broiler chickens colonized with *C. jejuni*. Dietary GOS modulated the immune response to *C. jejuni* by increasing cytokine IL-17A expression at colonization. In a study conducted in chicks by Sato et al. [29], an increase in IL-2 and IFN-γ mRNA expression in the foregut of 3- and 7-day-old chicks (but not in all experimental groups) was reported after dietary probiotic supplementation. Sławińska et al. [35] assessed the impact of synbiotics injected in ovo on cytokine gene expression (IL-4, IL-6, IL-8, IL-12, IL-18, IFN-β) in 6-week-old chickens. They found that gene expression of all cytokines in cecal tonsils was downregulated (except for IL-18). Contrary to that, upregulation of gene expression of IL-4, IL-6, IFN-β, and IL-18 was observed in the spleen (expression of IL-12 and IFN-γ was downregulated). These results confirmed that the effects of bioactive compounds on the lymphocyte activity depend on the lymphatic organs, which was also confirmed in our study. Contradiction with the results obtained by other authors may be due to slightly different research conditions, i.e., differences in preparation, administration route, etc.

The mechanism of the immunomodulatory action of probiotics as well as prebiotics and synbiotics is not fully understood. However, it is known that interaction between beneficial gut microbiota, including probiotics, is pivotal to mucosal tissue homeostasis and innate immunity [36,37]. Epithelial cells in the gastrointestinal tract create a first line of host defence against harmful microorganisms. Due to modulation of the phosphorylation of cytoskeletal and tight junction proteins, certain probiotics can improve cell–cell interactions and the stability of this barrier [36,38]. The gut microbiota and the interventions improving it (e.g., probiotics, prebiotics, synbiotics) are probably the most important factors for gut immune system development [39]. This may be especially important in chickens because they have a well-developed intestinal immune system (gut-associated lymphoid tissue—GALT) [40].

## 5. Conclusions

In ovo administration of prebiotics and synbiotics is a promising approach for enhancing chicken immune system functions. The increase in TCRγδ^+^ T cells observed after in ovo administration of inulin with *Lactococcus lactis* subsp. *lactis* IBB SL1 (Syn1) and Bi^2^tos with *Lactococcus lactis* subsp. *cremoris* IBB SC1 (Syn2) is worth highlighting. In light of our study, it is likely that the tested prebiotic and synbiotic containing inulin (Pre1 and Syn1) may stimulate the humoral immune response of broiler chickens. These effects may be valuable regarding chicken immune responses during infection or after vaccination. Based on the results discussed, we conclude that a combination of inulin and *Lactococcus lactis* subsp. *lactis* IBB SL1 was the most effective of the tested substances in the stimulation of the chicken immune system.

## Figures and Tables

**Table 1:** The schedule of the experiment.

Day of Experiment	Groups Injected In Ovo with Bioactive Components	Control Group	Pre1 Group	Pre2 Group	Syn1 Group	Syn2 Group	Procedure
		Numbers of Chickens/Groups					
0		49	49	49	49	49	
7 first immunization	Non-immunized	−7	−7	−7	−7	−7	chickens randomly selected and euthanized
	Non-immunized	14	14	14	14	14	chickens randomly selected
	SRBC-immunized	14	14	14	14	14	chickens randomly selected and immunized
	DEX-immunized	14	14	14	14	14	chickens randomly selected and immunized
21 second immunization	Non- immunized	−7	−7	−7	−7	−7	chickens randomly selected and euthanized
	SRBC-immunized	−7	−7	−7	−7	−7	chickens randomly selected and euthanized
	DEX-immunized	−7	−7	−7	−7	−7	chickens randomly selected and euthanized
	Non- immunized	7	7	7	7	7	chickens randomly selected
	SRBC-immunized	7	7	7	7	7	chickens randomly selected and immunized
	DEX-immunized	7	7	7	7	7	chickens randomly selected and immunized
35	Non- immunized	−7	−7	−7	−7	−7	euthanized chickens
	SRBC-immunized	−7	−7	−7	−7	−7	euthanized chickens
	DEX-immunized	−7	−7	−7	−7	−7	euthanized chickens

## Data Availability

Data is contained within Supplementary Materials.

## Supplementary Materials

The following are available online at https://www.mdpi.com/2076-2615/11/2/476/s1, Table S1: The percentage (%) of lymphocyte subsets in the bursa of Fabricius of 7-day-old chicken broilers after in ovo injection of the investigated prebiotics or synbiotics. Table S2: The percentage (%) of lymphocyte subsets in the bursa of Fabricius of 21-day-old chicken broilers after in ovo injection of the investigated prebiotics or synbiotics. Table S3: The total count (×10^6^) of lymphocyte subsets in the bursa of Fabricius of 21-day-old chicken broilers after in ovo injection of the investigated prebiotics or synbiotics. Table S4: The percentage (%) of lymphocyte subsets in the bursa of Fabricius of 35-day-old chicken broilers after in ovo injection of the investigated prebiotics or synbiotics. Table S5: The total count (×10^6^) of lymphocyte subsets in the bursa of Fabricius of 35-day-old chicken broilers after in ovo injection of the investigated prebiotics or synbiotics. Table S6: The percentage (%) of lymphocyte subsets in the thymus of 21-day-old chicken broilers after in ovo injection of the investigated prebiotics or synbiotics. Table S7: The total count (×10^6^) of lymphocyte subsets in the thymus of 21-day-old chicken broilers after in ovo injection of the investigated prebiotics or synbiotics. Table S8: The percentage (%) of lymphocyte subsets in the thymus of 35-day-old chicken broilers after in ovo injection of the investigated prebiotics or synbiotics. Table S9: The total count (×10^6^) of lymphocyte subsets in the thymus of 35-day-old chicken broilers after in ovo injection of the investigated prebiotics or synbiotics. Table S10: The percentage (%) of lymphocyte subsets in the spleen of 7-day-old chicken broilers after in ovo injection of the investigated prebiotics or synbiotics. Table S11: The total count (×10^6^) of lymphocyte subsets in the spleen of 7-day-old chicken broilers after in ovo injection of the investigated prebiotics or synbiotics. Table S12: The percentage (%) of lymphocyte subsets in the spleen of 21-day-old chicken broilers after in ovo injection of the investigated prebiotics or synbiotics. Table S13: The total count (×10^6^) of lymphocyte subsets in the spleen of 21-day-old chicken broilers after in ovo injection of the investigated prebiotics or synbiotics. Table S14: The percentage (%) of lymphocyte subsets in the spleen of 35-day-old chicken broilers after in ovo injection of the investigated prebiotics or synbiotics. Table S15: The total count (×10^6^) of lymphocyte subsets in the spleen of 35-day-old chicken broilers after in ovo injection of the investigated prebiotics or synbiotics.
