# Supplementary material for: Effects of Selected Prebiotics or Synbiotics Administered in ovo on Lymphocyte Subsets in Bursa of the Fabricius, Thymus, and Spleen in Non-Immunized and Immunized Chicken Broilers
Source: Animals (Basel). 2021 Feb 11;11(2):476. doi: 10.3390/ani11020476 (PMC7917990; doi:10.3390/ani11020476)
Supplement: Supplementary file 1 [file animals-11-00476-s001.pdf]

# Supplementary Materials: Effects of Selected Prebiotics or Synbiotics Administered *in ovo* on Lymphocyte Subsets in Bursa of Fabricius, Thymus and Spleen in Non-Immunized and Immunized Chicken Broilers

**Marianna Szczypka, Agnieszka Suszko-Pawłowska, Maciej Kuczkowski, Michał Gorczykowski, Magdalena Lis, Artur Kowalczyk, Ewa Łukaszewicz, Dominik Poradowski, Iwona Zbyryt, Marek Bednarczyk and Tadeusz Stefaniak**

**Table S1.** The percentage (%) of lymphocyte subsets in the bursa of Fabricius of 7-day-old chicken broilers after *in ovo* injection of the investigated prebiotics or synbiotics. The values are presented as mean ± S.D.; $n = 7$.Data with different superscript letters show significant differences ($p < 0.05$).

| Group | Bu-1$^{+}$ | CD3$^{+}$ |
|---|---|---|
| **CONTROL** | 98.09 $^{a}$ ± 0.96 | 0.84 $^{a}$ ± 0.36 |
| **PREBIOTIC 1** | 98.33 $^{a}$ ± 1.01 | 0.51 $^{ab}$ ± 0.30 |
| **PREBIOTIC 2** | 98.79 $^{a}$ ± 0.51 | 0.50 $^{ab}$ ± 0.13 |
| **SYNBIOTIC 1** | 98.59 $^{a}$ ± 0.53 | 0.82 $^{a}$ ± 0.40 |
| **SYNBIOTIC 2** | 99.08 $^{a}$ ± 0.25 | 0.32 $^{b}$ ± 0.09 |

**Table S2.** The percentage (%) of lymphocyte subsets in the bursa of Fabricius of 21-day-old chicken broilers after *in ovo* injection of the investigated prebiotics or synbiotics. The values are presented as mean ± S.D.; $n = 7$. Data with different superscript letters show significant differences ($p < 0.05$).

| Group | | Bu-1$^{+}$ | CD3$^{+}$ |
|---|---|---|---|
| **N0N-IMMUNIZED** | **CONTROL** | 96.99 $^{a}$ ± 0.53 | 1.27 $^{a}$ ± 0.27 |
| | **PREBIOTIC 1** | 93.87 $^{a}$ ± 6.05 | 1.22 $^{a}$ ± 0.41 |
| | **PREBIOTIC 2** | 96.67 $^{a}$ ± 2.22 | 1.40 $^{a}$ ± 0.89 |
| | **SYNBIOTIC 1** | 95.14 $^{a}$ ± 1.61 | 1.77 $^{a}$ ± 0.78 |
| | **SYNBIOTIC 2** | 96.45 $^{a}$ ± 1.90 | 1.14 $^{a}$ ± 0.36 |
| **SRBC-IMMUNIZED** | **CONTROL** | 97.10 $^{a}$ ± 0.87 | 1.34 $^{a}$ ± 0.82 |
| | **PREBIOTIC 1** | 90.37 $^{a}$ ± 8.31 | 1.45 $^{a}$ ± 0.33 |
| | **PREBIOTIC 2** | 96.46 $^{a}$ ± 1.34 | 1.40 $^{a}$ ± 0.37 |
| | **SYNBIOTIC 1** | 94.67 $^{a}$ ± 2.85 | 1.90 $^{a}$ ± 1.17 |
| | **SYNBIOTIC 2** | 96.04 $^{a}$ ± 1.93 | 1.28 $^{a}$ ± 0.39 |

| | | | |
|---|---|---|---|
| **DEX-IMMUNIZED** | **CONTROL** | 97.32 $^{a}$ ± 0.56 | 1.30 $^{a}$ ± 0.55 |
| | **PREBIOTIC 1** | 91.86 $^{b}$ ± 5.28 | 1.41 $^{a}$ ± 0.43 |
| | **PREBIOTIC 2** | 94.94 $^{ab}$ ± 2.71 | 1.87 $^{a}$ ± 0.85 |
| | **SYNBIOTIC 1** | 97.03 $^{a}$ ± 1.25 | 1.02 $^{a}$ ± 0.36 |
| | **SYNBIOTIC 2** | 97.21 $^{ab}$ ± 1.89 | 1.00 $^{a}$ ± 0.21 |

**Table S3.** The total count (x $10^{6}$) of lymphocyte subsets in the bursa of Fabricius of 21-day-old chicken broilers after *in ovo* injection of the investigated prebiotics or synbiotics. The values are presented as mean ± S.D.; $n = 7$. Data with different superscript letters show significant differences ($p < 0.05$).

| **Group** | | **Bu-1$^{+}$** | **CD3$^{+}$** |
|---|---|---|---|
| **N0N-IMMUNIZED** | **CONTROL** | 726.19 $^{a}$ ± 180.06 | 9.44 $^{a}$ ± 2.95 |
| | **PREBIOTIC 1** | 1041.59 $^{a}$ ± 350.64 | 14.90 $^{a}$ ± 11.19 |
| | **PREBIOTIC 2** | 1111.61 $^{a}$ ± 302.90 | 14.39 $^{a}$ ± 5.86 |
| | **SYNBIOTIC 1** | 1091.03 $^{a}$ ± 292.89 | 20.30 $^{a}$ ± 10.24 |
| | **SYNBIOTIC 2** | 880.21 $^{a}$ ± 228.65 | 10.05 $^{a}$ ± 2.60 |
| **SRBC-IMMUNIZED** | **CONTROL** | 918.94 $^{a}$ ± 184.15 | 13.53 $^{a}$ ± 10.05 |
| | **PREBIOTIC 1** | 997.43 $^{a}$ ± 257.93 | 15.39 $^{a}$ ± 2.66 |
| | **PREBIOTIC 2** | 1003.55 $^{a}$ ± 430.22 | 13.76 $^{a}$ ± 4.75 |
| | **SYNBIOTIC 1** | 1011.77 $^{a}$ ± 193.13 | 22.56 $^{a}$ ± 18.60 |
| | **SYNBIOTIC 2** | 1066.20 $^{a}$ ± 373.68 | 15.54 $^{a}$ ± 11.45 |
| **DEX-IMMUNIZED** | **CONTROL** | 1075.55 $^{a}$ ± 314.32 | 14.53 $^{a}$ ± 7.59 |
| | **PREBIOTIC 1** | 1081.75 $^{a}$ ± 333.10 | 18.02 $^{a}$ ± 9.92 |
| | **PREBIOTIC 2** | 1169.17 $^{a}$ ± 384.27 | 25.61 $^{a}$ ± 22.12 |
| | **SYNBIOTIC 1** | 1040.66 $^{a}$ ± 356.22 | 10.56 $^{a}$ ± 5.18 |
| | **SYNBIOTIC 2** | 976.69 $^{a}$ ± 241.55 | 10.17 $^{a}$ ± 3.41 |

**Table S4.** The percentage (%) of lymphocyte subsets in the bursa of Fabricius of 35-day-old chicken broilers after *in ovo* injection of the investigated prebiotics or synbiotics. The values are presented as mean ± S.D.; $n = 7$. Data with different superscript letters show significant differences ($p < 0.05$).

| Group | | Bu-1⁺ | CD3⁺ |
|---|---|---|---|
| NON-IMMUNIZED | CONTROL | 96.75 [a] ± 0.74 | 1.01 [a] ± 0.39 |
| | PREBIOTIC 1 | 96.67 [ab] ± 1.00 | 1.11 [a] ± 0.40 |
| | PREBIOTIC 2 | 94.48 [ab] ± 1.93 | 1.29 [a] ± 0.53 |
| | SYNBIOTIC 1 | 93.23 [b] ± 3.60 | 1.48 [a] ± 0.55 |
| | SYNBIOTIC 2 | 94.27 [ab] ± 2.62 | 1.71 [a] ± 0.70 |
| SRBC-IMMUNIZED | CONTROL | 96.48 [a] ± 1.17 | 1.22 [ab] ± 0.34 |
| | PREBIOTIC 1 | 97.15 [a] ± 0.53 | 0.94 [a] ± 0.26 |
| | PREBIOTIC 2 | 92.48 [ab] ± 1.61 | 1.21 [ab] ± 0.36 |
| | SYNBIOTIC 1 | 86.83 [b] ± 7.71 | 3.75 [b] ± 3.68 |
| | SYNBIOTIC 2 | 96.15 [a] ± 3.30 | 1.06 [a] ± 0.49 |
| DEX-IMMUNIZED | CONTROL | 94.97 [a] ± 3.49 | 1.23 [a] ± 0.24 |
| | PREBIOTIC 1 | 96.86 [a] ± 0.94 | 1.31 [a] ± 0.65 |
| | PREBIOTIC 2 | 90.45 [a] ± 3.40 | 1.34 [a] ± 0.43 |
| | SYNBIOTIC 1 | 93.82 [a] ± 3.51 | 0.95 [a] ± 0.29 |
| | SYNBIOTIC 2 | 94.55 [a] ± 5.52 | 1.61 [a] ± 0.80 |

**Table S5.** The total count (x $10^6$) of lymphocyte subsets in the bursa of Fabricius of 35-day-old chicken broilers after *in ovo* injection of the investigated prebiotics or synbiotics. The values are presented as mean ± S.D.; *n* = 7. Data with different superscript letters show significant differences (*p* < 0.05).

| Group | | Bu-1+ | CD3+ |
|---|---|---|---|
| N0N-IMMUNIZED | CONTROL | 1903.85 [a] ± 806.36 | 20.68 [a] ± 13.03 |
| | PREBIOTIC 1 | 2529.71 [a] ± 711.40 | 27.97 [a] ± 9.57 |
| | PREBIOTIC 2 | 4466.72 [a] ± 4024.52 | 50.62 [a] ± 29.06 |
| | SYNBIOTIC 1 | 4911.93 [a] ± 3996.01 | 86.17 [a] ± 82.45 |
| | SYNBIOTIC 2 | 3659.75 [a] ± 2340.16 | 54.95 [a] ± 23.71 |
| SRBC-IMMUNIZED | CONTROL | 2294.20 [a] ± 753.55 | 27.68 [a] ± 6.97 |
| | PREBIOTIC 1 | 3232.92 [a] ± 1770.65 | 30.74 [a] ± 17.65 |
| | PREBIOTIC 2 | 5865.91 [a] ± 5017.82 | 70.86 [ab] ± 62.13 |
| | SYNBIOTIC 1 | 5166.53 [a] ± 3769.91 | 148.24 [b] ± 91.66 |
| | SYNBIOTIC 2 | 3687.07 [a] ± 2151.14 | 33.57 [a] ± 14.59 |
| DEX-IMMUNIZED | CONTROL | 2731.67 [a] ± 973.78 | 32.47 [a] ± 13.33 |
| | PREBIOTIC 1 | 4343.64 [a] ± 2527.67 | 56.62 [a] ± 44.43 |
| | PREBIOTIC 2 | 3958.61 [a] ± 1935.41 | 55.98 [a] ± 30.67 |
| | SYNBIOTIC 1 | 5662.96 [a] ± 3996.53 | 63.37 [a] ± 56.21 |
| | SYNBIOTIC 2 | 3544.82 [a] ± 1802.13 | 57.46 [a] ± 25.58 |

**Table S6.** The percentage (%) of lymphocyte subsets in the thymus of 21-day-old chicken broilers after *in ovo* injection of the investigated prebiotics or synbiotics. The values are presented as mean ± S.D.; $n = 7$. Data with different superscript letters show significant differences ($p < 0.05$).

| | Group | $CD3^{+}$ $TCR\gamma\delta^{+}$ | $CD4^{-}8^{-}$ | $CD4^{+}8^{+}$ | $CD4^{+}$ | $CD8^{+}$ | $CD4^{+}/CD8^{+}$ |
|---|---|---|---|---|---|---|---|
| NON-IMMUNIZED | CONTROL | $7.57^{a}$ ± 1.35 | $17.48^{a}$ ± 3.12 | $72.30^{ab}$ ± 2.58 | $4.69^{a}$ ± 1.35 | $5.53^{a}$ ± 1.55 | $0.95^{a}$ ± 0.51 |
| | PREBIOTIC 1 | $10.84^{ab}$ ± 6.01 | $15.54^{a}$ ± 4.75 | $70.56^{ab}$ ± 5.78 | $5.90^{a}$ ± 2.35 | $7.99^{b}$ ± 1.33 | $0.74^{a}$ ± 0.27 |
| | PREBIOTIC 2 | $9.84^{ac}$ ± 3.93 | $12.98^{a}$ ± 5.49 | $77.54^{a}$ ± 7.57 | $3.50^{a}$ ± 0.85 | $5.98^{ab}$ ± 1.70 | $0.61^{a}$ ± 0.14 |
| | SYNBIOTIC 1 | $13.72^{bc}$ ± 4.46 | $19.28^{a}$ ± 5.50 | $66.21^{b}$ ± 6 .13 | $5.51^{a}$ ± 1.56 | $6.77^{ab}$ ± 1.46 | $0.83^{a}$ ± 0.29 |
| | SYNBIOTIC 2 | $16.62^{b}$ ± 1.04 | $17.92^{a}$ ± 2.19 | $70.83^{ab}$ ± 3.14 | $5.30^{a}$ ± 0.88 | $5.95^{ab}$ ± 0.55 | $0.89^{a}$ ± 0.15 |
| SRBC-IMMUNIZED | CONTROL | $8.05^{a}$ ± 2.09 | $17,44^{a}$ ± 3.92 | $71.60^{a}$ ± 4.47 | $4.79^{a}$ ± 1.33 | $6.16^{a}$ ± 1.58 | $0.82^{a}$ ± 0.27 |
| | PREBIOTIC 1 | $7.77^{a}$ ± 3.88 | $13.20^{a}$ ± 2.45 | $71.47^{a}$ ± 5.24 | $5.63^{a}$ ± 1.49 | $7.23^{a}$ ± 1.81 | $0.73^{a}$ ± 0.26 |
| | PREBIOTIC 2 | $12.29^{ab}$ ± 5.24 | $13.73^{a}$ ± 5.73 | $74.45^{a}$ ± 8.65 | $5.57^{a}$ ± 1.93 | $6.24^{a}$ ± 1.74 | $0.92^{a}$ ± 0.32 |
| | SYNBIOTIC 1 | $15.24^{b}$ ± 6.07 | $18.80^{a}$ ± 5.88 | $67.27^{a}$ ± 7.71 | $6.27^{a}$ ± 1.97 | $7.65^{a}$ ± 1.61 | $0.87^{a}$ ± 0.43 |
| | SYNBIOTIC 2 | $15.05^{b}$ ± 2.50 | $19.13^{a}$ ± 3.46 | $68.16^{a}$ ± 6.59 | $6.27^{a}$ ± 3.53 | $6.46^{a}$ ± 1.31 | $0.95^{a}$ ± 0.46 |
| DEX-IMMUNIZED | CONTROL | $7.27^{a}$ ± 1.42 | $16.34^{a}$ ± 2.15 | $73.45^{a}$ ± 2.65 | $4.68^{a}$ ± 0.48 | $5.52^{a}$ ± 1.05 | $0.87^{a}$ ± 1.16 |
| | PREBIOTIC 1 | $7.09^{a}$ ± 2.66 | $16.02^{a}$ ± 4.55 | $72.06^{a}$ ± 5.64 | $5.00^{a}$ ± 0.71 | $6.91^{a}$ ± 1.07 | $0.74^{a}$ ± 0.15 |
| | PREBIOTIC 2 | $11.11^{ab}$ ± 4.65 | $16.34^{a}$ ± 6.36 | $72.15^{a}$ ± 10.76 | $4.84^{a}$ ± 2.68 | $6.66^{a}$ ± 2.47 | $0.75^{a}$ ± 0.34 |
| | SYNBIOTIC 1 | $14.32^{b}$ ± 4.97 | $20.55^{a}$ ± 3.27 | $65.08^{a}$ ± 5.97 | $7.23^{a}$ ± 2.68 | $7.14^{a}$ ± 0.55 | $1.02^{a}$ ± 0.36 |
| | SYNBIOTIC 2 | $16.51^{b}$ ± 3.07 | $20.98^{a}$ ± 6.98 | $66.83^{a}$ ± 6.74 | $5.97^{a}$ ± 4.43 | $6.21^{a}$ ± 1.92 | $0.90^{a}$ ± 0.41 |

**Table S7.** The total count (x $10^6$) of lymphocyte subsets in the thymus of 21-day-old chicken broilers after *in ovo* injection of the investigated prebiotics or synbiotics. The values are presented as mean ± S.D.; $n = 7$. Data with different superscript letters show significant differences ($p < 0.05$).

| | Group | $CD3^+$ $TCR\gamma\delta^+$ | $CD4^-8^-$ | $CD4^+8^+$ | $CD4^+$ | $CD8^+$ |
|---|---|---|---|---|---|---|
| NON-IMMUNIZED | CONTROL | 127.21 [a] ± 43.65 | 297.59 [a] ± 123.31 | 1239.10 [a] ± 500.14 | 76.80 [a] ± 26.65 | 95.13 [a] ± 47.65 |
| | PREBIOTIC 1 | 225.96 [a] ± 137.51 | 315.04 [a] ± 137.83 | 1389.73 [a] ± 459.07 | 111.91 [a] ± 38.47 | 153.61 [a] ± 39.31 |
| | PREBIOTIC 2 | 218.96 [a] ± 86.08 | 285.65 [a] ± 103.13 | 1782.45 [a] ± 580.33 | 78.27 [a] ± 23.48 | 133.67 [a] ± 41.33 |
| | SYNBIOTIC 1 | 264.68 [a] ± 100.16 | 370.32 [a] ± 48.97 | 1341.80 [a] ± 381.34 | 97.10 [a] ± 17.77 | 136.38 [a] ± 40.38 |
| | SYNBIOTIC 2 | 339.87 [a] ± 46.08 | 370.45 [a] ± 38.74 | 1504.10 [a] ± 367.56 | 109.88 [a] ± 19.86 | 125.20 [a] ± 27.45 |
| SRBC-IMMUNIZED | CONTROL | 129.03 [a] ± 44.72 | 279.84 [a] ± 90.17 | 1137.07 [a] ± 237.14 | 76.21 [a] ± 25.96 | 101.74 [a] ± 44.00 |
| | PREBIOTIC 1 | 162.33 [a] ± 109.30 | 256.90 [a] ± 60.42 | 1401.25 [ab] ± 278.59 | 117.57 [a] ± 45.47 | 143.76 [a] ± 49.47 |
| | PREBIOTIC 2 | 440.96 [a] ± 473.66 | 659.75 [a] ± 772.97 | 2909.19 [b] ± 1960.89 | 268.98 [a] ± 316.98 | 250.01 [a] ± 191.42 |
| | SYNBIOTIC 1 | 325.07 [a] ± 109.53 | 425.14 [a] ± 60.62 | 1632.15 [ab] ± 518.86 | 145.02 [a] ± 40.37 | 181.58 [a] ± 53.07 |
| | SYNBIOTIC 2 | 306.64 [a] ± 66.71 | 375.94 [a] ± 72.79 | 1463.19 [ab] ± 493.49 | 129.47 [a] ± 72.88 | 133.83 [a] ± 37.31 |
| DEX-IMMUNIZED | CONTROL | 112.35 [a] ± 29.23 | 252.77 [a] ± 68.87 | 1121.58 [a] ± 174.92 | 72.26 [a] ± 16.57 | 85.37 [a] ± 22.39 |
| | PREBIOTIC 1 | 160.72 [a] ± 100.82 | 326.50 [a] ± 43.60 | 1580.33 [ab] ± 517.11 | 107.07 [a] ± 28.34 | 146.66 [ab] ± 34.33 |
| | PREBIOTIC 2 | 439.49 [a] ± 563.14 | 710.78 [a] ± 753.90 | 2773.70 [b] ± 1646.37 | 219.21 [a] ± 267.26 | 285.84 [b] ± 283.82 |
| | SYNBIOTIC 1 | 282.47 [a] ± 55.97 | 431.44 [a] ± 108.07 | 1466.96 [ab] ± 636.29 | 144.47 [a] ± 35.75 | 155.56 [ab] ± 54.13 |
| | SYNBIOTIC 2 | 317.82 [a] ± 28.81 | 383.48 [a] ± 95.08 | 1276.26 [ab] ±389.07 | 108.00 [a] ± 72.65 | 115.24 [ab] ± 31.65 |

**Table S8.** The percentage (%) of lymphocyte subsets in the thymus of 35-day-old chicken broilers after *in ovo* injection of the investigated prebiotics or synbiotics. The values are presented as mean ± S.D.; $n = 7$. Data with different superscript letters show significant differences ($p < 0.05$).

| | Group | CD3 + TCRγδ+ | CD4-8- | CD4 + 8+ | CD4+ | CD8+ | CD4 + / CD8+ |
|---|---|---|---|---|---|---|---|
| NON-IMMUNIZED | CONTROL | 9.41 [a] ± 3.96 | 17.06 [a] ± 4.12 | 71.17 [a] ± 4.42 | 5.05 [a] ± 0.95 | 6.72 [a] ± 1.12 | 0.78 [ab] ± 0.22 |
| | PREBIOTIC 1 | 6.64 [a] ± 0.64 | 12.00 [b] ± 1.30 | 78.08 [b] ± 2.76 | 5.10 [a] ± .,19 | 4.81 [bc] ± 0.93 | 1.09 [a] ± 0.31 |
| | PREBIOTIC 2 | 5.93 [a] ± 2.02 | 17.63 [a] ± 3,31 | 73.49 [ab] ± 2.84 | 4.34 [a] ± 1.08 | 4.54 [b] ± 1.04 | 0.95 [ac] ± 0.06 |
| | SYNBIOTIC 1 | 10.94 [a] ± 4.21 | 16.34 [ab] ± 1.80 | 72.67 [ab] ± 2.56 | 3.77 [a] ± 1.13 | 7.22 [ac] ± 0.76 | 0.52 [b] ± 0.15 |
| | SYNBIOTIC 2 | 10.01 [a] ± 4.48 | 14.17 [ab] ± 3.66 | 75.66 [ab] ± 5.66 | 4.21 [a] ± 1.08 | 5.97 [ab] ± 1.68 | 0.73 [bc] ± 0.16 |
| SRBC-IMMUNIZED | CONTROL | 7.85 [ab] ± 1.31 | 21.15 [a] ± 4.93 | 66.78 [a] ± 4.98 | 5.64 [a] ± 1.29 | 6.44 [a] ± 0.71 | 0.90 [a] ± 0.27 |
| | PREBIOTIC 1 | 6.12 [a] ± 1.41 | 10.37 [b] ± 2.16 | 81.14 [b] ± 2.63 | 3.44 [a] ± 0.69 | 5.04 [a] ± 1.12 | 0.73 [a] ± 0.31 |
| | PREBIOTIC 2 | 6.85 [ab] ± 0.80 | 18.25 [a] ± 5.57 | 71.49 [a] ± 7.22 | 4.55 [a] ± 1.41 | 5.38 [a] ± 1.78 | 0.89 [a] ± 0.25 |
| | SYNBIOTIC 1 | 11.19 [bc] ± 4.84 | 16.10 [ab] ± 4.88 | 71.86 [a] ± 7.13 | 5.39 [a] ± 2.32 | 6.64 [a] ± 1.34 | 0.80 [a] ± 0.30 |
| | SYNBIOTIC 2 | 12.32 [c] ± 3.59 | 17.77 [a] ± 3.40 | 70.38 [a] ± 3.63 | 4.88 [a] ± 0.79 | 6.98 [a] ± 1.12 | 0.71 [a] ± 0.15 |
| DEX-IMMUNIZED | CONTROL | 8.11 [ab] ± 1.31 | 18.22 [a] ± 2.75 | 70.69 [a] ±2.55 | 4.79 [ab] ± 1.13 | 6.30 [ab] ± 1.30 | 0.77 [a] ± 0.15 |
| | PREBIOTIC 1 | 8.56 [ab] ± 2.92 | 14.75 [a] ± 7.16 | 76.71 [a] ± 8.62 | 3.74 [ab] ± 0.77 | 4.78 [ab] ± 1.08 | 0.81 [a] ± 0.20 |
| | PREBIOTIC 2 | 6.76 [a] ± 1.32 | 20.14 [a] ± 4.55 | 71.71 [a] ± 5.37 | 3.64 [a] ± 0.52 | 4.52 [a] ± 1.02 | 0.83 [a] ± 0.19 |
| | SYNBIOTIC 1 | 7.45 [a] ± 2.43 | 16.40 [a] ± 2.20 | 75.22 [a] ± 2.60 | 3.58 [a] ± 1.02 | 4.80 [ab] ± 1.30 | 0.83 [a] ± 0.44 |
| | SYNBIOTIC 2 | 11.53 [b] ± 3.69 | 16.92 [a] ± 5.22 | 70.93 [a] ± 6.62 | 5.22 [b] ± 0.31 | 6.91 [b] ± 1.36 | 0.78 [a] ± 0.15 |

**Table S9.** The total count (x 10[6]) of lymphocyte subsets in the thymus of 35-day-old chicken broilers after *in ovo* injection of the investigated prebiotics or synbiotics. The values are presented as mean ± S.D.; $n = 7$. Data with different superscript letters show significant differences ($p < 0.05$).

| | Group | $CD3^+$ $TCR\gamma\delta^+$ | $CD4^-8^-$ | $CD4^+8^+$ | $CD4^+$ | $CD8^+$ |
|---|---|---|---|---|---|---|
| NON-IMMUNIZED | CONTROL | 351.05 [a] ± 116.60 | 678.13 [a] ± 302.00 | 2801.07 [a] ± 868.32 | 200.42 [a] ± 80.83 | 268.87 [a] ± 98.33 |
| | PREBIOTIC 1 | 464.72 [a] ± 157.71 | 851.26 [a] ± 325.65 | 5480.81 [a] ± 1731.16 | 370.91 [a] ± 177.56 | 331.19 [a] ± 118.20 |
| | PREBIOTIC 2 | 427.98 [a] ± 223.00 | 1177.41 [a] ± 451.11 | 4856.52 [a] ± 1545.93 | 292.19 [a] ± 136.62 | 300.66 [a] ± 124.08 |
| | SYNBIOTIC 1 | 517.23 [a] ± 201.25 | 874.13 [a] ± 334.87 | 4055.60 [a] ± 1972.60 | 219.01 [a] ± 152.28 | 391.26 [a] ± 173.06 |
| | SYNBIOTIC 2 | 412.05 [a] ± 199.99 | 630.79 [a] ± 303.70 | 3242.42 [a] ± 844.35 | 183.28 [a] ± 75.54 | 257.19 [a] ± 106.30 |
| SRBC-IMMUNIZED | CONTROL | 384.57 [a] ± 228.29 | 1007.61 [a] ± 637.92 | 3114.08 [a] ± 1480.70 | 259.76 [a] ± 137.28 | 298.49 [a] ± 150.11 |
| | PREBIOTIC 1 | 489.31 [a] ± 175.85 | 844.66 [a] ± 326.36 | 7077.35 [a] ± 3649.37 | 296.07 [a] ± 145.33 | 410.44 [a] ± 172.46 |
| | PREBIOTIC 2 | 462.15 [a] ± 163.89 | 1255.81 [a] ± 646.71 | 4817.20 [a] ± 1609.86 | 302.56 [a] ± 125.02 | 359.16 [a] ± 161.47 |
| | SYNBIOTIC 1 | 851.14 [a] ± 587.07 | 1272.22 [a] ± 1038.57 | 4902.02 [a] ± 2379.67 | 406.65 [a] ± 323.49 | 472.53 [a] ± 291.75 |
| | SYNBIOTIC 2 | 623.41 [a] ± 273.37 | 916.14 [a] ± 370.69 | 3526.22 [a] ± 1016.67 | 256.20 [a] ± 114.30 | 354.87 [a] ± 133.17 |
| DEX-IMMUNIZED | CONTROL | 284.23 [a] ± 96.87 | 674.28 [a] ± 199.30 | 2605.39 [a] ± 544.96 | 173.98 [a] ± 41.51 | 226.48 [a] ± 41.71 |
| | PREBIOTIC 1 | 856.77 [b] ± 758.22 | 1339.95 [a] ± 887.93 | 6833.19 [b] ± 3979.88 | 346.93 [a] ± 231.63 | 422.66 [a] ± 243.16 |
| | PREBIOTIC 2 | 417.85 [ab] ± 104.73 | 1341.32 [a] ± 313.30 | 4943.33 [ab] ± 1552.18 | 247.50 [a] ± 67.69 | 295.03 [a] ± 26.68 |
| | SYNBIOTIC 1 | 423.72 [ab] ± 180.76 | 914.68 [a] ± 186.86 | 4259.68 [ab] ± 1127.00 | 205.48 [a] ± 89.50 | 260.16 [a] ± 58.99 |
| | SYNBIOTIC 2 | 677.92 [ab] ± 290.07 | 1076.67 [a] ± 551.37 | 4485.92 [ab] ± 1639.59 | 335.15 [a] ± 137.23 | 445.76 [a] ± 223.72 |

**Table S10.** The percentage (%) of lymphocyte subsets in the spleen of 7-day-old chicken broilers after *in ovo* injection of the investigated prebiotics or synbiotics. The values are presented as mean ± S.D.; $n = 7$.Data with different superscript letters show significant differences ($p < 0.05$).

| Group | Bu-1+ | CD3+ | CD3+ TCRγδ+ | CD3+ CD4+ | CD3+ CD8+ | CD4+/ CD8+ |
|---|---|---|---|---|---|---|
| CONTROL | 10.41 ab ± 4.99 | 79.79 a ± 3.52 | 8.24 ab ± 1.49 | 31.89 a ± 7.99 | 44.94 a ± 4.02 | 0.73 a ± 0.24 |
| PREBIOTIC 1 | 9.44 a ± 2.32 | 75.05 a ± 4.01 | 9.57 ab ±2.05 | 25.71 ab ± 5.57 | 45.36 a ± 4.65 | 0.58 a ± 1.19 |
| PREBIOTIC 2 | 16.66 ab ± 6.96 | 64.94 b ± 7,69 | 10.24 a ± 1.79 | 23.99 ab ± 4.38 | 39.03 a ± 6.30 | 0.62 a ± 0.12 |
| SYNBIOTIC 1 | 18.74 b ± 7,86 | 59.63 b ± 6.35 | 6.84 b ± 1.91 | 22.32 b ± 3.10 | 38.37 a ± 9.87 | 0.64 a ± 0.26 |
| SYNBIOTIC 2 | 14.62 ab ± 2.29 | 65.99 b ± 3.34 | 8.02 ab ± 1.70 | 27.52 ab ± 4.98 | 37.66 a ± 5.09 | 0.75 a ± 0.24 |

**Table S11.** The total count (x $10^6$) of lymphocyte subsets in the spleen of 7-day-old chicken broilers after *in ovo* injection of the investigated prebiotics or synbiotics. The values are presented as mean ± S.D.; $n = 7$. Data with different superscript letters show significant differences ($p < 0.05$).

| Group | CD4+8+ | CD3+ | CD3+ TCRγδ+ | CD3+ CD4+ | CD3+ CD8+ |
|---|---|---|---|---|---|
| CONTROL | 18.28 a ± 10.27 | 134.89 a ± 26.33 | 13.85 a ± 3.39 | 54.94 a ± 21.03 | 75.70 a ± 14.80 |
| PREBIOTIC 1 | 10.74 a ± 3.27 | 84.87 b ± 14.26 | 11.02 a ± 3.67 | 29.46 b ± 9.64 | 50.93 b ± 7.42 |
| PREBIOTIC 2 | 21.48 a ± 10.26 | 80.95 b ± 11.90 | 12.86 a ± 3.00 | 29.67 b ± 4.33 | 48.83 b ± 10.38 |
| SYNBIOTIC 1 | 21.82 a ± 8.52 | 70.41 b ± 10.48 | 8.10 a ± 2.56 | 26.13 b ± 3.03 | 45.58 b ± 13.17 |
| SYNBIOTIC 2 | 20.53 a ± 7.67 | 93.84 b ± 33.38 | 11.82 a ± 5.42 | 37.63 a ± 11.68 | 55.17 a ± 23.08 |

**Table S12.** The percentage (%) of lymphocyte subsets in the spleen of 21-day-old chicken broilers after *in ovo* injection of the investigated prebiotics or synbiotics. The values are presented as mean ± S.D.; $n = 7$. Data with different superscript letters show significant differences ($p < 0.05$).

| | Group | Bu-1⁺ | CD3⁺ | CD3⁺ TCRγδ⁺ | CD3⁺ CD4⁺ | CD3⁺ CD8⁺ | CD4⁺/ CD8⁺ |
|---|---|---|---|---|---|---|---|
| NON-IMMUNIZED | CONTROL | 17.07 [a] ± 5.12 | 72.58 [a] ± 6.77 | 13.97 [ab] ± 2.57 | 25.02 [a] ± 4.13 | 42.99 [ab] ± 6.57 | 0.59 [a] ± 0.08 |
| | PREBIOTIC 1 | 23.66 [b] ± 3.54 | 61.13 [b] ± 7.87 | 11.41 [a] ± 2.94 | 26.27 [a] ± 4.56 | 37.81 [ac] ± 2.59 | 0.70 [a] ± 0.14 |
| | PREBIOTIC 2 | 21.25 [ab] ± 3.84 | 67.39 [ab] ± 4.77 | 14.06 [ab] ± 2.26 | 24.58 [a] ± 2.54 | 44.53 [b] ± 3.27 | 0.55 [a] ± 0.08 |
| | SYNBIOTIC 1 | 16.59 [ab] ± 0.80 | 74.88 [a] ± 2.57 | 16.98 [b] ± 2.28 | 30.87 [a] ± 3.07 | 34.07 [c] ± 1.58 | 0.90 [a] ± 0.07 |
| | SYNBIOTIC 2 | 20.85 [ab] ± 4.88 | 63.96 [ab] ± 7.28 | 15.40 [ab] ± 2.11 | 24.46 [a] ± 11.79 | 34.13 [c] ± 4.61 | 0.74 [a] ± 0.41 |
| SRBC-IMMUNIZED | CONTROL | 24.06 [a] ± 5.88 | 63.43 [a] ± 7.44 | 13.70 [a] ± 2.51 | 20.99 [a] ± 3.47 | 38.65 [a] ± 8.62 | 0.56 [a] ± 0.15 |
| | PREBIOTIC 1 | 19.93 [a] ± 2.07 | 65.03 [a] ± 7.57 | 11.83 [a] ± 1.87 | 26.22 [a] ± 4.22 | 37.08 [a] ± 4.33 | 0.71 [a] ± 0.16 |
| | PREBIOTIC 2 | 20.86 [a] ± 5.34 | 65.55 [a] ± 8.92 | 12.59 [a] ± 2.59 | 25.76 [a] ± 7.24 | 39.45 [a] ± 6.23 | 0.67 [a] ± 0.23 |
| | SYNBIOTIC 1 | 14.87 [a] ± 1.20 | 75.46 [a] ± 1.68 | 14.35 [a] ± 4.28 | 32.30 [a] ± 7.97 | 38.31 [a] ± 9.08 | 0.90 [a] ± 0.39 |
| | SYNBIOTIC 2 | 22.92 [a] ± 5.60 | 65.92 [a] ± 5.17 | 13.14 [a] ± 3.46 | 24.04 [a] ± 7.44 | 38.18 [a] ± 4.53 | 0.66 [a] ± 0.13 |
| DEX-IMMUNIZED | CONTROL | 23.70 [a] ± 7.90 | 62.78 [a] ± 10.30 | 10.16 [a] ± 1.17 | 20.07 [a] ± 6.06 | 38.52 [a] ± 13.28 | 0.62 [a] ± 0.29 |
| | PREBIOTIC 1 | 17.39 [a] ± 4.53 | 64.98 [a] ± 6.00 | 11.64 [a] ± 2.84 | 23.54 [a] ± 3.73 | 39.88 [a] ± 6.33 | 0.60 [a] ± 0.14 |
| | PREBIOTIC 2 | 18.04 [a] ± 4.21 | 71.53 [a] ± 5.84 | 13.31 [ab] ± 3.75 | 24.36 [a] ± 2.35 | 43.43 [a] ± 6.24 | 0.58 [a] ± 0.07 |
| | SYNBIOTIC 1 | 21.94 [a] ± 7.17 | 62.97 [a] ± 6.95 | 17.92 [b] ± 2.28 | 25.89 [a] ± 2.30 | 39.64 [a] ± 5.82 | 0.66 [a] ± 0.06 |
| | SYNBIOTIC 2 | 21.21 [a] ± 5.79 | 62.16 [a] ± 7.19 | 10.56 [a] ± 2.65 | 24.24 [a] ± 4.97 | 38.16 [a] ± 6.24 | 0.66 [a] ± 0.22 |

**Table S13.** The total count (x $10^6$) of lymphocyte subsets in the spleen of 21-day-old chicken broilers after *in ovo* injection of the investigated prebiotics or synbiotics. The values are presented as mean ± S.D.; $n = 7$. Data with different superscript letters show significant differences ($p < 0.05$).

| | Group | $Bu\text{-}1^+$ | $CD3^+$ | $CD3^+$ $TCR\gamma\delta^+$ | $CD3^+$ $CD4^+$ | $CD3^+$ $CD8^+$ |
|---|---|---|---|---|---|---|
| NON-IMMUNIZED | CONTROL | 92.58 [a] ± 55.05 | 367.40 [a] ± 105.12 | 72.18 [a] ± 30.99 | 131.42 [a] ± 39.75 | 214.49 [a] ± 49.97 |
| | PREBIOTIC 1 | 156.31 [a] ± 47.65 | 405.62 [a] ± 133.83 | 70.84 [a] ± 9.71 | 169.46 [a] ± 41.80 | 250.28 [ab] ± 76.75 |
| | PREBIOTIC 2 | 175.15 [a] ± 46.12 | 553.45 [a] ± 87.97 | 116.06 [ab] ± 28.05 | 202.45 [a] ± 39.14 | 368.21 [b] ± 74.11 |
| | SYNBIOTIC 1 | 142.62 [a] ± 16.76 | 644.04 [a] ± 76.41 | 156.88 [b] ± 25.04 | 266.30 [a] ± 46.152 | 293.09 [ab] ± 36.86 |
| | SYNBIOTIC 2 | 160.06 [a] ± 25.98 | 502.44 [a] ± 104.43 | 120.48 [ab] ± 25.12 | 193.50 [a] ± 107.25 | 260.70 [ab] ± 50.92 |
| SRBC-IMMUNIZED | CONTROL | 175.81 [a] ± 74.57 | 451.40 [a] ± 136.91 | 96.93 [a] ± 29.94 | 146.61 [a] ± 36.59 | 271.90 [a] ± 76.25 |
| | PREBIOTIC 1 | 138.46 [a] ± 21.30 | 455.33 [a] ± 91.12 | 81.73 [a] ± 12.24 | 181.29 [a] ± 43.22 | 259.46 [a] ± 53.57 |
| | PREBIOTIC 2 | 179.27 [a] ± 89.77 | 535.81 [a] ± 138.50 | 105.64 [a] ± 42.31 | 210.70 [a] ± 69.78 | 320.71 [a] ± 73.46 |
| | SYNBIOTIC 1 | 113.58 [a] ± 13.59 | 579.21 [a] ± 58.48 | 108.18 [a] ± 31.68 | 250.81 [a] ± 78.28 | 290.01 [a] ± 44.09 |
| | SYNBIOTIC 2 | 185.36 [a] ± 59.01 | 535.36 [a] ± 105.34 | 109.61 [a] ± 41.37 | 203.60 [a] ± 91.23 | 316.49 [a] ± 73.25 |
| DEX-IMMUNIZED | CONTROL | 140.64 [a] ± 58.29 | 378.35 [a] ± 108.56 | 61.32 [a] ± 17.11 | 119.84 [a] ± 47.36 | 232.27 [a] ± 106.27 |
| | PREBIOTIC 1 | 179.38 [a] ± 64.55 | 686.61 [a] ± 228.82 | 122.94 [b] ± 44.48 | 234.04 [a] ± 112.29 | 371.86 [ab] ± 120.75 |
| | PREBIOTIC 2 | 154.65 [a] ± 26.32 | 652.17 [a] ± 258.42 | 116.85 [ab] ± 36.71 | 219.48 [a] ± 74.19 | 399.18 [b] ± 99.69 |
| | SYNBIOTIC 1 | 197.80 [a] ± 100.15 | 536.78 [a] ± 105.70 | 147.44 [b] ± 42.98 | 216.64 [a] ± 60.99 | 325.47 [ab] ± 69.58 |
| | SYNBIOTIC 2 | 150.54 [a] ± 35.58 | 449.62 [a] ± 93.72 | 77.68 [ab] ± 31.30 | 172.29 [a] ± 49.80 | 274.09 [ab] ± 79.69 |

**Table S14.** The percentage (%) of lymphocyte subsets in the spleen of 35-day-old chicken broilers after *in ovo* injection of the investigated prebiotics or synbiotics. The values are presented as mean ± S.D.; $n = 7$. Data with different superscript letters show significant differences ($p < 0.05$).

| | Group. | Bu-1$^+$ | CD3$^+$ | CD3$^+$ TCRγδ$^+$ | CD3$^+$ CD4$^+$ | CD3$^+$ CD8$^+$ | CD4$^+$/ CD8$^+$ |
|---|---|---|---|---|---|---|---|
| NON-IMMUNIZED | CONTROL | 20.17$^a$ ± 5.89 | 73.25$^a$ ± 6.81 | 12.84$^a$ ± 2.69 | 24.93$^a$ ± 2.32 | 46.61$^a$ ± 4.77 | 0.54$^a$ ± 0.07 |
| | PREBIOTIC 1 | 36.72$^b$ ± 10.95 | 53.54$^b$ ± 12.12 | 14.73$^{ab}$ ± 3.30 | 20.83$^a$ ± 6.59 | 29.84$^b$ ± 7.98 | 0.72$^{ab}$ ± 0.18 |
| | PREBIOTIC 2 | 19.32$^a$ ± 7.50 | 70.10$^{ac}$ ± 11.88 | 15.01$^{ab}$ ± 2.55 | 28.99$^a$ ± 4.25 | 35.14$^{bc}$ ± 7.10 | 0.84$^b$ ± 0.14 |
| | SYNBIOTIC 1 | 24.68$^a$ ± 4.45 | 55.60$^{bc}$ ± 13.31 | 15.52$^{ab}$ ± 3.29 | 21.49$^a$ ± 6.79 | 34.02$^{bc}$ ± 7.96 | 0.60$^{ab}$ ± 0.11 |
| | SYNBIOTIC 2 | 18.36$^a$ ± 4.06 | 72.53$^a$ ± 5.15 | 21.36$^b$ ± 8.00 | 30.05$^a$ ± 8.31 | 41.40$^{ac}$ ± 6.54 | 0.74$^{ab}$ ± 0.27 |
| SRBC-IMMUNIZED | CONTROL | 19.56$^a$ ± 3.43 | 73.60$^a$ ± 4.96 | 13.85$^a$ ± 1.67 | 25.56$^a$ ± 3.56 | 40.89$^a$ ± 5.28 | 0.63$^{ab}$ ± 0.09 |
| | PREBIOTIC 1 | 22.81$^a$ ± 6.21 | 64.24$^a$ ± 15.61 | 13.01$^a$ ± 2.38 | 26.22$^a$ ± 8.17 | 32.67$^a$ ± 9.32 | 0.81$^a$ ± 0.17 |
| | PREBIOTIC 2 | 22.83$^a$ ± 5.76 | 66.35$^a$ ± 8.15 | 14.37$^a$ ± 4.49 | 25.59$^a$ ± 3.09 | 35.71$^a$ ± 6.48 | 0.74$^{ab}$ ± 0.18 |
| | SYNBIOTIC 1 | 36.58$^b$ ± 6.37 | 45.19$^b$ ± 14.59 | 15.64$^a$ ± 3.71 | 14.55$^b$ ± 5.93 | 30.56$^a$ ± 10.45 | 0.50$^b$ ± 0.17 |
| | SYNBIOTIC 2 | 21.04$^a$ ± 3.68 | 68.24$^a$ ± 6.30 | 20.65$^a$ ± 8.84 | 26.61$^a$ ± 5.27 | 41.10$^a$ ± 2.32 | 0.65$^{ab}$ ± 0.14 |
| DEX-IMMUNIZED | CONTROL | 24.45 ± 3.15 | 67.88$^a$ ± 4.55 | 12.74$^a$ ± 2.28 | 25.09$^a$ ± 7.57 | 39.67$^{ab}$ ± 4.57 | 0.64$^a$ ± 0.21 |
| | PREBIOTIC 1 | 25.18 ± 7.88 | 62.59$^a$ ± 4.18 | 13.66$^a$ ± 2.66 | 25.59$^a$ ± 10.35 | 29.80$^{ab}$ ± 8.51 | 0.85$^a$ ± 0.29 |
| | PREBIOTIC 2 | 28.41 ± 7.57 | 57.43$^{ab}$ ± 7.15 | 14.30$^a$ ± 3.02 | 21.81$^a$ ± 7.26 | 29.14$^a$ ± 6.25 | 0.74$^a$ ± 0.36 |
| | SYNBIOTIC 1 | 24.26 ± 7.80 | 43.18$^b$ ± 18.63 | 13.61$^a$ ± 2.69 | 20.74$^a$ ± 7.95 | 30.55$^{ab}$ ± 12.37 | 0.72$^a$ ± 0.23 |
| | SYNBIOTIC 2 | 19.25 ± 5.66 | 69.16$^a$ ± 6.71 | 27.21$^b$ ± 11.55 | 22.78$^a$ ± 4.62 | 41.73$^b$ ± 5.59 | 0.55$^a$ ± 0.14 |

**Table S15.** The total count (x $10^6$) of lymphocyte subsets in the spleen of 35-day-old chicken broilers after *in ovo* injection of the investigated prebiotics or synbiotics.The values are presented as mean ± S.D.; $n = 7$ . Data with different superscript letters show significant differences ($p < 0.05$).

| | Title | Bu-1$^+$ | CD3$^+$ | CD3$^+$ TCRγδ$^+$ | CD3$^+$ CD4$^+$ | CD3$^+$ CD8$^+$ |
|---|---|---|---|---|---|---|
| NON-IMMUNIZED | CONTROL | 672.56 $^{ab}$ ± 188.23 | 2640.87 $^a$ ±1164.22 | 450.45 $^a$ ± 172.35 | 884.56 $^a$ ± 324.12 | 1686.70 $^a$ ± 775.12 |
| | PREBIOTIC 1 | 1295.50 $^a$ ± 858.45 | 1734.88 $^a$ ± 516.38 | 478.28 $^a$ ± 149.67 | 686.40 $^a$ ± 263.51 | 929.55 $^a$ ± 180.67 |
| | PREBIOTIC 2 | 693.18 $^{ab}$ ± 477.43 | 2352.54 $^a$ ± 747.86 | 510.26 $^a$ ± 209.12 | 958.08 $^a$ ± 251.62 | 1156.68 $^a$ ± 353.06 |
| | SYNBIOTIC 1 | 800.79 $^{ab}$ ± 278.61 | 1749.34 $^a$ ± 472.16 | 482.08 $^a$ ± 79.59 | 653.09 $^a$ ± 208.59 | 1074.61 $^a$ ± 297.88 |
| | SYNBIOTIC 2 | 565.54 $^b$ ± 220.15 | 2174.21 $^a$ ± 364.37 | 635.16 $^a$ ± 213.29 | 868.42 $^a$ ± 116.23 | 1244.63 $^a$ ± 278.13 |
| SRBC-IMMUNIZED | CONTROL | 552.97 $^a$ ± 218.53 | 2090.05 $^a$ ± 718.72 | 391.40 $^a$ ± 145.22 a | 737.48 $^a$ ± 303.66 | 1187.08 $^a$ ± 486.32 |
| | PREBIOTIC 1 | 755.65 $^{ab}$ ± 242.82 | 2214.44 $^a$ ± 985.31 | 434.74 $^a$ ± 153.17 | 885.98 $^a$ ± 355.71 | 1150.65 $^a$ ± 612.84 |
| | PREBIOTIC 2 | 633.57 $^a$ ± 161.76 | 1926.43 $^a$ ± 636.13 | 428.81 $^a$ ± 216.91 | 727.10 $^a$ ± 176.43 | 1052.39 $^a$ ± 429.12 |
| | SYNBIOTIC 1 | 1442.58 $^b$ ± 607.34 | 1921.43 $^a$ ± 1221.36 | 612.21 $^a$ ± 221.71 | 597.55 $^a$ ± 358.83 | 1327.30 $^a$ ± 941.32 |
| | SYNBIOTIC 2 | 672.57 $^a$ ± 118.48 | 2181.81 $^a$ ± 199.45 | 679.96 $^a$ ± 368.85 | 845.87 $^a$ ± 122.66 | 1321.56 $^a$ ± 165.27 |
| DEX-IMMUNIZED | CONTROL | 887.11 $^a$ ± 383.58 | 2528.90 $^a$ ± 1187.92 | 448.71 $^a$ ± 162.24 | 980.43 $^a$ ± 602.76 | 1500.46 $^a$ ± 811.18 |
| | PREBIOTIC 1 | 1038.02 $^a$ ± 204.01 | 2734.04 $^a$ ± 934.31 | 583.12 $^{ab}$ ± 154.45 | 1130.28 $^a$ ± 521.05 | 1315.43 $^a$ ± 547.13 |
| | PREBIOTIC 2 | 792.79 $^a$ ± 185.91 | 1741.68 $^a$ ± 712.23 | 412.48 $^a$ ± 122.09 | 703.25 $^a$ ± 205.31 | 901.67 $^a$ ± 426.65 |
| | SYNBIOTIC 1 | 862.89 $^a$ ± 341.16 | 1516.51 $^a$ ± 781.13 | 477.73 $^{ab}$ ± 152.50 | 718.29 $^a$ ± 317.24 | 1066.50 $^a$ ± 490.19 |
| | SYNBIOTIC 2 | 592.87 $^a$ ± 159.35 | 2196.58 $^a$ ± 580.81 | 844.07 $^b$ ± 375.91 | 713.72 $^a$ ± 185.84 | 1334.86 $^a$ ± 413.59 |
